# Leptin modulated microRNA-628-5p targets Jagged-1 and inhibits prostate cancer hallmarks

**DOI:** 10.1038/s41598-022-13279-x

**Published:** 2022-06-16

**Authors:** Leslimar Rios-Colon, Juliet Chijioke, Suryakant Niture, Zainab Afzal, Qi Qi, Anvesha Srivastava, Malathi Ramalinga, Habib Kedir, Patrice Cagle, Elena Arthur, Mitu Sharma, John Moore, Gagan Deep, Simeng Suy, Sean P. Collins, Deepak Kumar

**Affiliations:** 1 Julius L. Chambers Biomedical/Biotechnology Research Institute (BBRI), North Carolina Central University, 1801 Fayetteville St., Durham, NC 27707 USA; 2 Department of Cancer Biology, Wake Forest Baptist Medical Center, Winston-Salem, NC 27157, USA; 3 Wake Forest Baptist Comprehensive Cancer Center, Wake Forest Baptist Medical Center, Winston-Salem, NC 27157, USA; 4 Lombardi Comprehensive Cancer Center, Georgetown University, Washington, 20057 USA

**Keywords:** Cancer, Cell biology

## Abstract

MicroRNAs (miRNAs) are single-stranded non-coding RNA molecules that play a regulatory role in gene expression and cancer cell signaling. We previously identified miR-628-5p (miR-628) as a potential biomarker in serum samples from men with prostate cancer (PCa) (Srivastava et al. in Tumour Biol 35:4867–4873, 10.1007/s13277-014-1638-1, 2014). This study examined the detailed cellular phenotypes and pathways regulated by miR-628 in PCa cells. Since obesity is a significant risk factor for PCa, and there is a correlation between levels of the obesity-associated hormone leptin and PCa development, here we investigated the functional relationship between leptin and miR-628 regulation in PCa. We demonstrated that exposure to leptin downregulated the expression of miR-628 and increased cell proliferation/migration in PCa cells. We next studied the effects on cancer-related phenotypes in PCa cells after altering miR-628 expression levels. Enforced expression of miR-628 in PCa cells inhibited cell proliferation, reduced PCa cell survival/migration/invasion/spheroid formation, and decreased markers of cell stemness. Mechanistically, miR-628 binds with the *JAG1*-3′UTR and inhibits the expression of Jagged-1 (JAG1). JAG1 inhibition by miR-628 downregulated Notch signaling, decreased the expression of Snail/Slug, and modulated epithelial-mesenchymal transition and invasiveness in PC3 cells. Furthermore, expression of miR-628 in PCa cells increased sensitivity towards the drugs enzalutamide and docetaxel by induction of cell apoptosis. Collectively our data suggest that miR-628 is a key regulator of PCa carcinogenesis and is modulated by leptin, offering a novel therapeutic opportunity to inhibit the growth of advanced PCa.

## Introduction

Prostate cancer (PCa) is the most commonly diagnosed non-cutaneous cancer and the second leading cause of cancer deaths in men, accounting for over 248,530 estimated new cases and 34,130 deaths in 2021^1^. PCa mortality is mainly driven by metastatic castration-resistant prostate cancer (mCRPC) which is defined by progression to metastatic disease coupled with the development of resistance to anti-androgen therapies^2–4^. PCa is also the most commonly diagnosed cancer in African American (AA) men, accounting for nearly one-third of cancers diagnosed in this population and characterized by worse PCa-specific outcomes compared to Caucasian American (CA) men^5^. Disparities in the prognosis of advanced disease could be explained by social determinants, such as support mechanisms and access to health care, and by variations in tumor phenotypes^6^. Obesity is also a factor, as obese men have an increased risk of developing advanced PCa disease^7^, and obesity has been strongly associated with increased PCa risk among AA men^8^.

Due to the ubiquitous nature of adipose tissue, many types of solid tumors grow in close or direct contact with adipocytes and other adipose-associated cell populations^9^. The specific nature of the reciprocal communication occurring between a developing tumor and adjacent adipose tissue is an area of active study. A growing body of literature indicates that interactions with the local adipose milieu are an important driver of PCa malignancy. Adipocytes exhibit both short and long-range interaction with cancer cells and can regulate gene expression in other peripheral tissues through the secretion of adipokines. Adipokines, such as leptin,  insulin-like growth factor 1 (IGF1), interleukin-6 (IL-6), and vascular endothelial growth factor (VEGF), are upregulated in obese populations and demonstrated to interfere with several signaling pathways and exert mitogenic effects^9^. Leptin, in particular, is a major adipose cytokine that controls body weight by regulating energy intake and expenditure, and clinical studies have demonstrated a positive correlation between blood leptin levels and PCa development^10^.

Evidence suggests that differences in low-grade disease and patient outcomes are also driven by underlying tumor genomic differences, including overexpression or suppression of microRNAs (miRNAs)^11^. MiRNAs bind 3′UTRs (untranslated regions), and it is estimated that 60% of protein-coding genes have predicted miRNAs-binding sites^12^. MiRNAs have critical regulatory roles in gene expression affecting many cellular processes, including growth, proliferation, differentiation, metabolism, and cell death^12^**.** Aberrant expression and dysregulation of miRNAs have been associated with various human diseases, including cancer, autoimmune diseases, and cardiovascular and neurological disorders^13^. Bioinformatics analysis indicates that a single 3′UTR can be targeted by several miRNAs^14–16^. This important characteristic of miRNA biology is called ‘genomic redundancy’ since a miRNA is predicted to repress the expression of thousands of mRNAs, and each mRNA could be targeted by different miRNAs^14^. Due to their relevance in numerous diseases, including cancers, and their stability in various bio-fluids, they offer a novel opportunity to serve as prognostic and diagnostic biomarkers to identify cancer development and progression^7,15^. Interestingly, the complicated crosstalk between obese-related adipokines, such as leptin, includes a post-transcriptional gene regulation and an altered miRNA transcriptome, which can play a role in tumor proliferation, invasion, apoptosis, and angiogenesis^17^.

MiR-628-5p (miR-628) is located at the 15q21.3 chromosome region and commonly acts as a tumor suppressor^18^. MiR-628 has frequently been implicated as a tumor suppressor in various cancers; however, a few studies have also suggested its involvement in oncogenesis^19^. Our group was the first to report that miR-628 is differentially expressed in serum obtained from PCa patients compared to serum obtained from healthy individuals^20^. Differential expression levels of miR-628 were validated through RT/qPCR, with levels significantly lower in PCa patients when compared to healthy individuals. Decreased expression of miR-628 was observed in both AA and CA PCa populations compared to matched controls, emphasizing the discriminatory power and relevance of this miRNA in PCa^20^. The miR-628 expression has also been reported to reduce PCa cell proliferation by targeting the fibroblast growth factor receptor 2 (FGFR2) signaling pathway^21^.

The present study explored the association of adipokine leptin and miRNA-628 regulation and their contribution to PCa development/suppression. In addition, we investigated the effect of leptin in the modulation of miR-628 expression and PCa cell aggressiveness. We have demonstrated that overexpression of miR-628 in PCa cells inhibited cell proliferation/migration/invasion/spheroid formation by targeting Jagged1, suggesting that miR-628 acts tumor suppressor.

## Materials and methods

### Cell culture and treatments

PCa cell lines DU145, PC3, 22Rv1, MDA-PCa-2B and LNCaP were purchased from the American Type Culture Collection (ATCC) (Cat# HTB-81, Cat# CRL-1435, Cat# CRL-2505, Cat# CRL-2422 and Cat# CRL-1740 respectively). Epithelial prostate cell line RWPE1 was obtained from ATCC (Cat# CRL-11609). DU145, PC3, 22Rv1, and LNCaP cells were cultured in Roswell Park Memorial Institute (RPMI) medium (Life Technologies) supplemented with 10% heat-inactivated fetal bovine serum (FBS) and 1% penicillin/streptomycin. MDA-PCa-2b cells were cultured in human prostate cell 1 (HPC1) medium (Cat# 0403, AthenaES) supplemented with 20% heat-inactivated FBS and 1% penicillin/streptomycin. To promote MDA-PCa-2b attachment, cell culture plates were pre-coated with poly-l-lysine (50 μg/ml). RWPE-1 cells were grown in Keratinocyte Serum-Free Medium (K-SFM) supplemented with human recombinant Epidermal Growth Factor 1–53 (EGF 1–53) and Bovine Pituitary Extract (BPE) and 1% penicillin/streptomycin (Cat# 17005-042, Gibco,). Cells were maintained in a humidified cell culture incubator with 5% CO_2_ at 37 °C.

Human leptin (Cat# Z02962-200, GenScript) was reconstituted in water following the manufacturer’s instructions. Before leptin treatment, cells were grown in serum-free media for 24 h, and then cells were treated with increasing concentrations of leptin. The effect of leptin on miR-628 expression was analyzed. Jagged-1 peptide (Cat #188-204), Notch ligand peptide (Cat# AS-61298), and a Jagged-1 scrambled control (Cat# AS-64239) were obtained from Anaspec (Ca, USA). Both peptides were reconstituted and utilized according to the manufacturer’s instructions. In some experiments, cells were exposed to enzalutamide (Selleckchem; Cat. # S1250) and docetaxel (Selleckchem; Cat. # No. S1148) after transfection with miR-628 or negative control (NC), and the effect of miR-628 on drug sensitivity was determined.

### MiR-628 mimic and negative control mimic (NC) transfection

PCa cells (~ 70% confluence) were transiently transfected with 100 nM of mirVana™ miRNA mimic miR-628 (Assay ID: MC12826, Life Technologies) or *mir*Vana™ miRNA mimic, negative control #1 (NC) (Life Technologies, Cat#4464058) using Lipofectamine-2000 transfection reagent (Cat# 11668019, Life Technologies) following manufacturer’s specifications. Transfection efficiency was also confirmed by utilizing Cy3-labeled miR-628 mimic or NC control (Bioneer, Oakland, CA) and after visualization of PCa cells with a fluorescence microscope.

### Western Blotting

Western blotting was performed as described previously^22^. Briefly, cell lysates from all PCa cells were prepared using a lysis buffer (Cell Signaling Technology, Danvers, MA, USA) containing a protease inhibitor cocktail (Roche, Indianapolis, IN, USA). To isolate nuclear and cytosolic protein fractions, we utilized the Nuclear Extract Kit (Cat# 40010, Active Motif) following the manufacturer’s protocol. Protein concentration was determined using the Bio-Rad Bradford reagent (Bio-Rad, Hercules, CA, USA). Equal amounts of protein (60 µg) were separated using NU-PAGE 4–12% Bis–Tris gels (Cat# NP0321BOX, Invitrogen) and then transferred onto a polyvinylidene difluoride (PVDF) membrane (Millipore, Billerica, MA). Membranes were blocked in casein blocking buffer (1×) (Sigma-Aldrich, St. Louis, MO). In some cases, membranes were cut before hybridization using the molecular weight marker as a reference. Membranes were then probed individually overnight at 4 °C with each corresponding primary antibody. The following commercially available antibodies were obtained from Cell Signaling Technology: anti-Jagged-1 (Cat# 2155S), anti-Hes-1 (Cat #11988S), anti-Notch1(Cat #3608S), anti-Notch2 (Cat #5732S), anti-Notch3 (Cat #5276S), anti-E-cadherin (Cat #3195S), anti-N-cadherin (Cat #13116S), anti-PARP (Cat #9542P), anti-cleaved Caspase-3 (Cat #9664S), anti-Snail (Cat #3879S), anti-Slug (Cat# 9585S), anti-Nanog (Cat#4903S), anti-Vimentin (Cat# 5741S0, anti-NICD (Cat#4147S), anti-CD44 (Cat# 37259), anti- zeb1(Cat #3396), anti-β-Tubulin (Cat # 2128T) and anti-GAPDH (Cat #5174S). Membranes were washed 3 times, 5 min each with TBST, and then incubated with respective secondary antibodies conjugated with horseradish peroxidase (HRP) for one hour at room temperature. After washing, the membranes were processed using a chemiluminescence (ECL) system, and immunoblot signals were captured using an Azure C-500 Bio-system imager or X-ray films. The band intensities were quantified by utilizing the gels analyzing feature in ImageJ (version 1.53 k, https://imagej.nih.gov/ij/). Western blot images were cropped to highlight specific bands.

### RT/qPCR

Total RNA from RWPE1 and PCa cell lines was isolated using TRIZOL reagent (Cat# 15596026, Life Technologies). One microgram of RNA was reverse transcribed using a High Capacity cDNA Reverse Transcription kit (Cat# 4368814, Applied Biosystems). After cDNA synthesis, one microliter of cDNA was incubated with Power SYBR Green PCR master mix (Cat# 4367659, Applied Biosystems) and mixed with indicated forward and reverse primers (Supplementary Table﻿ 1). GAPDH primers were used as an internal control. For miR-628 expression analysis, total microRNAs from normal RWPE1 and PCa cells were isolated using the mirVana microRNA Isolation Kit (Thermo Fisher Scientific). Total miRNAs (10 ng) were reverse transcribed using primers specific for miR-628 and RNU44 (Assay IDs 002433 and 001094, respectively, Applied Biosystems, Carlsbad, CA, USA) and TaqMan Reverse Transcription reagents (Applied Biosystems). Expression of miR-628 and RNU44 was quantified by RT/qPCR using TaqMan fast advance PCR master mixture and Taqman expression assay primers. RNU44 expression was used as an internal control and subtracted from miR-628 expression. The PCR reactions were run on a QuantStudio-3 PCR system (Applied Biosystems), and relative quantification (RQ) values were determined relative to the endogenous control (RNU44) according to the manufacturer's protocols.

### Analysis of miR-628 expression in PCa tumor samples

RNA samples from human PCa tissues (n = 24) and adjacent benign tissues (n = 24) were obtained from the Prostate Cancer Biorepository Network (PCBN) at Johns Hopkins University School of Medicine in Baltimore, MD, USA. Analysis of miR-628 expression was performed by reverse transcription using 10 ng of RNA and primers specific for miR-628 (Assay ID: 4464084) and RNU44 endogenous control (Assay ID: 001094). Expression of miR-628 and RNU44 was quantified by RT/qPCR as described in the above section.

### *JAG1* expression in PCa samples

Dataset GSE41969 was accessed through the Gene Expression Omnibus (GEO) (https://www.ncbi.nlm.nih.gov/geo/). This dataset comprises normal adjacent tissue with no discernible tumor upon pathology review (benign) and PCa samples obtained from AA and CA men^23^. Differentially expressed genes were identified using the GEO2R web tool (https://www.ncbi.nlm.nih.gov/geo/geo2r/) by comparing subgroups in this data set. *JAG1* expression data was obtained using the profile graph feature that shows the expression values of a specific gene across samples. This expression value was then analyzed using GraphPad Prism 9.

### Luciferase assay

LNCaP and PC3 PCa cells (1 × 10^4^ cells/well) were transfected with 0.5 µg of *JAG1*-3′UTR-Luciferase construct (OriGene). After 18 h, cells were co-transfected with NC mimic (100 nM) or miR-628 mimic (100 nM), miR-628 mutant-type mimic (designed from Integrated DNA Technologies), or NC mimic separately for an additional 24 h. After transfection, cells were washed with PBS and mixed with luciferase substrate (Promega). Plates were incubated for 15 min at room temperature in the dark. Luciferase activity was quantified using a Fluostar Omega plate reader (BMG Lab Tech, Cary, NC).

### Cellular survival/proliferation assay

Cell survival was assessed using 3-(4,5-dimethylthiazol-2-yl)-2,5-diphenyltetrazolium bromide (MTT) reagent (Cat# M5655, Sigma). Briefly, cells were grown in 96 well plates, transfected with either NC or miR-628 for indicated periods,, and incubated with 10 µl/well MTT reagent (Stock: 5 mg/ml in PBS) for 2 h at 37 °C. Cells were washed with PBS, and formazan crystals were dissolved in 200 µl of dimethyl sulfoxide (DMSO), and plates were read at 570 nm using a Fluostar Omega plate reader (BMG Lab Tech, Cary, NC, USA).

Cell proliferation was assessed using an Incucyte Zoom system for live cell analysis. Cells (1.0–2.0 × 10^5^) were plated in 6-well plates overnight and then transfected with NC mimic (100 nM) or miR-628 mimic (100 nM) for 48 h. The transfected cells were then harvested and plated in 96-well plates (2 × 10^3^ cells/well). Cell growth was monitored utilizing an IncuCyte^®^ ZOOM Live-Cell Analysis System (Essen BiosScience, Ann Arbor, Michigan). Real-time cellular proliferation was performed by analyzing the occupied area (% confluence) of cell images collected every 2 h for 70–120 h.

### Cell colony formation assay

PCa cells were transiently transfected with NC mimic (100 nM) or miR-628 mimic (100 nM) and cultured in 6-well plates (1 × 10^3^ per well) in triplicates and allowed to grow for 7–10 days. Media was removed, colonies were carefully washed with 1X PBS, and then cells were fixed with cold methanol for 20 min at room temperature. Colonies were stained with 0.5% crystal violet (dissolved in methanol) for 30 min, colonies were washed with water and blue colonies were counted using ImageJ (https://imagej.nih.gov/ij/).

### Wound healing assay

Cells were cultured at a density of 1.5–2.0 × 10^5^ per well in 6-well plates in triplicates and transiently transfected with NC mimic (100 nM) or miR-628 mimic (100 nM). After 24 h, a vertical scratch was made using a pipet tip (200 µl). Cells were imaged at 0 and 18 h by using a phase-contrast Zeiss microscope. Wound length was measured utilizing the ZEN software in pixels, and the percentage of wound closure was calculated using the formula [(A_0_ – A_24_)/A_0_] × 100.

### Spheroid culture assay

Spheroid culture assay was performed as previously described^24^. In brief, PCa cells were transfected with NC or miR-628 and plated at a density of 2500 cells/well in triplicates in ultralow attachment six-well culture plates (Corning, St. Louis, MO, USA) and grown in stem cell media consisting of DMEM/F12 (Gibco, Cat# 21331020) media supplemented with B27 (Gibco, Cat#17504044) and N-2 (Gibco, Cat# 17502001). Spheres were visualized and counted 10 days after plating using a light microscope. Sphere area was measured using the Zeiss ZEN lite software.

### Angiogenesis assay

Human Umbilical Vein Endothelial Cells (HUVECs) were obtained from Gibco (Cat# C-003-5C) and grown according to the manufacturer’s instructions in Medium 200 (Cat# M-200-500, Life Technologies) supplemented with LVES (50X) Large Vessel Endothelial Supplement (Cat# A14608-01, Life Technologies). For the angiogenesis assay, 1.0 × 10^5^ cells/well were plated in 6 well plates and transfected the following day with NC mimic (100 nM) or miR-628 mimic (100 nM) as previously described. Cells were then plated in 24 well plates coated with Geltrex^®^ LDEV-Free Reduced Growth Factor Basement Membrane Matrix (Cat# A14132-02, Life Technologies) in triplicates, and tubes were allowed to form for 24 h. Tube number and length were counted utilizing ImageJ software.

### Migration assay

Permeable cell culture inserts (Thomas Scientific, Swedesboro, NJ, USA) were used to assess cell migration. Cells were transfected with NC mimic (100 nM) or miR-628 mimic (100 nM) and then starved in serum-free media for 24 h. After starvation, cells were trypsinized and suspended in serum-free media at a density of 0.5 × 10^6^ cells/ml. Then 300 µl of cell suspension was added to the interior of each insert (triplicates). Complete media with 10% serum was used as an attractant and added to the lower chamber. After 24-48 h of incubation, migrating cells at the bottom of the insert were fixed in 10% methanol and stained with 0.5% crystal violet solution. Inserts were submerged in PBS to wash residual stain and allowed to air dry. Cells were visualized under a light microscope and quantified alternatively by dissolving stained cells in 10% acetic acid. Equal amounts (100 µl/well) of dye/solute mixture were then transferred to a 96 well plate (duplicated per insert), and absorbance was determined at 562 nm using a Fluostar Omega plate reader (BMG Lab Tech, Cary, NC, USA).

### Invasion assay

Invasion assay was performed using permeable cell culture inserts (Thomas Scientific, Swedesboro, NJ) coated with 30 µl of Corning^®^ Matrigel^®^ Growth Factor Reduced (GFR) Basement Membrane Matrix (Corning, NY). Inserts were incubated for 30 min at 37 °C to allow the matrigel to solidify. We followed a procedure similar to the migration assay described above but utilized matrigel coated inserts.

### Statistical analysis

Statistical analysis was performed utilizing the SigmaStat 4.0 software and GraphPad Prism 9. To evaluate differences between experimental variables, we utilized a t-test or one-way or two-way ANOVAs with Tukey’s multiple comparisons test. Differences were considered statistically significant at p values equal to or below 0.05 (p < 0.05). Data is presented as the following: ns p > 0.05, *p < 0.05, **p < 0.01, or ***p < 0.001. Data was also plotted using GraphPad Prism 9.

## Results

### MiR-628 is downregulated in PCa patient tumors and the adipokine leptin regulates its expression in PCa cells

**Figure 1:**
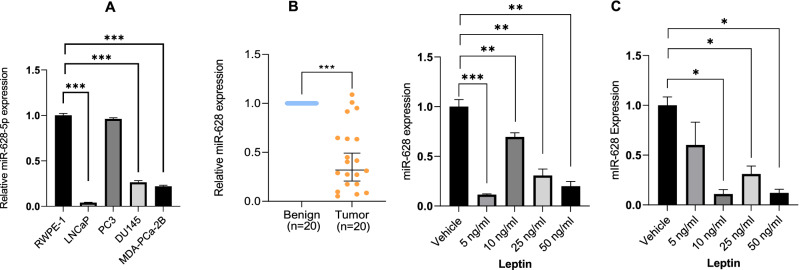
MiR-628 is downregulated in PCa patient tumors and adipokine leptin regulates its expression in PCa cells. (**A**) Basal expression of miR-628-5p was analyzed in a human epithelial prostate tissue cell line (derived from healthy non-cancerous tissue) RWPE-1 and in PCa cell lines (LNCaP, PC3, DU145, and MDA-PCa-2b) by RT/qPCR (***p < 0.001). (**B**) miR-628 expression was assessed in a cohort of PCa tissues (N = 20, blue) vs. benign adjacent (adj) control tissues (N = 20, orange) (***p < 0.001). (**C**) LNCaP and PC3 cells were treated with 5 ng/ml, 10 ng/ml, 25 ng/ml and 50 ng/ml of leptin or vehicle for 48 and the expression of miR-628 was analyzed by RT/qPCR (*p < 0.05, **p < 0.001, (***p < 0.001). Data represent average ± SEM. Experiments were performed in triplicates as described in the materials and methods section.

We first analyzed the basal expression of miR-628 in the human epithelial prostate tissue cell line RWPE-1 (derived from healthy non-cancerous tissue) and in various PCa cell lines of various lineages (LNCaP, PC3, DU145, and MDA-PCa-2b) by RT/qPCR (Fig. 1A). We observed lower expression of miR-628 in the PCa cell lines LNCaP, DU145, and MDA-PCa-2b compared to normal RWPE-1 cells (p < 0.001). However, the expression of miR-628 was not significantly changed between PC3 cells compared with normal prostate RWPE1 cells.

We previously demonstrated that miR-628 was downregulated in a subset of PCa patient sera compared to normal controls and that miR-628 levels could effectively discriminate between disease and non-diseased. We assessed miR-628 expression in a cohort of prostate cancer patient tissues compared to benign adjacent control tissues to further confirm these results. The expression of miR-628 was significantly downregulated (p < 0.0001) in the tumor samples compared to matched benign controls (Fig. 1B). Additionally, we assessed miR-628 levels utilizing the miRNA Target Viewer (miR-TV). This software utilizes the TCGA-PRAD (The Cancer Genome Atlas, Prostate Adenocarcinoma) dataset to show miRNA expression in 52 normal versus 494 tumor tissues^25^. TCGA-PRAD data revealed that miR-628 expression is significantly downregulated (p < 0.02) in patient tumor samples compared with the control group (Fig. 1S), suggesting that miR-628 expression is downregulated in PCa cells or tumors.

Due to the strong correlation between increased levels of adipokine leptin and PCa aggressiveness^10^, we analyzed the effect of leptin on miR-628 regulation. Interestingly, exposure to LNCaP and PC3 cells with 5 ng/ml, 10 ng/ml, 25 ng/ml, or 50 ng/ml of leptin for 48 h significantly decreased miR-628 expression in both LNCaP and PC3 cells (Fig. 1C, left and right panels). Together, our data indicated that miR-628 is downregulated in PCa tumor samples compared to normal matched controls, and the expression of miR-628 is controlled by adipokine leptin in PCa cells.

### Leptin abrogates miR-628 impact on cell proliferation/colony formation in PCa cells 

**Figure 2:**
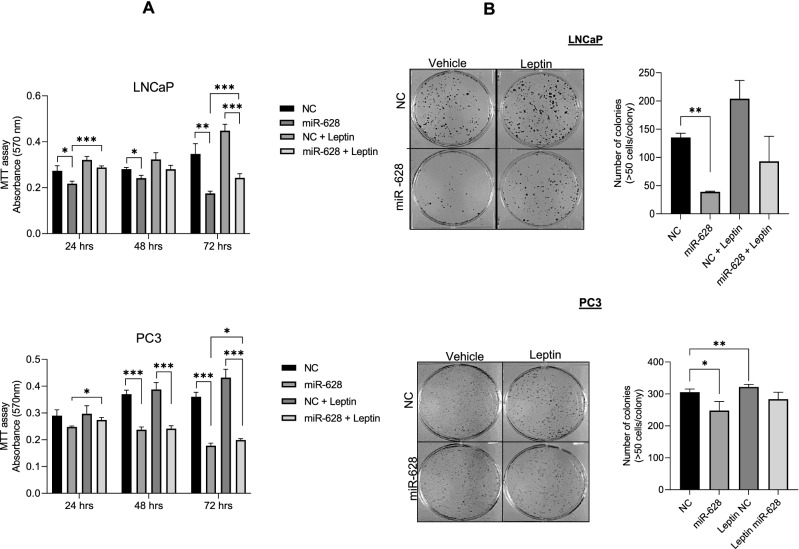
Leptin abrogates miR-628 impact on cell proliferation/colony formation in PCa cells. (**A**) LNCaP and PC3 cells were treated with 10 ng/ml of leptin or vehicle for 48 h and cell proliferation was analyzed using MTT assay in LNCaP and PC3 cells after transfection with 100 nM miR-628 mimic or NC and after treatment with 10 ng/ml of leptin or vehicle after 24, 48, and 72 h (upper and lower panels). *p < 0.05, **p < 0.01, ***p < 0.001. (**B**) Cell colony formation was assessed in LNCaP and PC3 cells transfected with 100 nM miR-628 mimic or NC and treated with 10 ng/ml or vehicle (left and right panels) *p < 0.05, **p < 0.01.

Since leptin exposure downregulated miR-628 expression in PCa cellular models, we assessed LNCaP and PC3 cell proliferation/survival after transfection with either NC or miR-628 and after treatment with leptin (10 ng/ml) (Fig. 2A). As expected, miR-628 overexpression decreased cell proliferation in both LNCaP and PC3 cells at all indicated time points (*p < 0.05, **p < 0.01, and ***p < 0.001). Transfection of NC and leptin treatments increased cell survival in LNCaP and PC3 cells when compared to miR-628 transfected cells. Interestingly, miR-628 expression and leptin treatment decreased cell proliferation compared with NC transfected and leptin treated cells, suggesting that leptin downregulates miR-628 expression and hence induced cell proliferation in PCa cells (Fig. 2A, upper and lower panels). Finally, the impact of leptin on PCa cell colony formation was determined in LNCaP and PC3. Our data further suggest that leptin treatment increased cell colony formation in LNCaP and PC3 cells (Fig. 2B, upper and lower panels). Transfection with miR-628 mimic and leptin treatments decreased cell colony formation, specifically in LNCaP cells indicating that leptin effectively decreases endogenous miR-628 expression and enhances cell colony formation (Fig. 2B upper and lower panels). In conclusion, the adipokine leptin downregulated miR-628 expression and increased cell proliferation/ cell colony formation and miR-628 overexpression partially abrogated the proliferative effects of this hormone.

### Overexpression of miR-628 affects PCa cell proliferation

**Figure 3:**
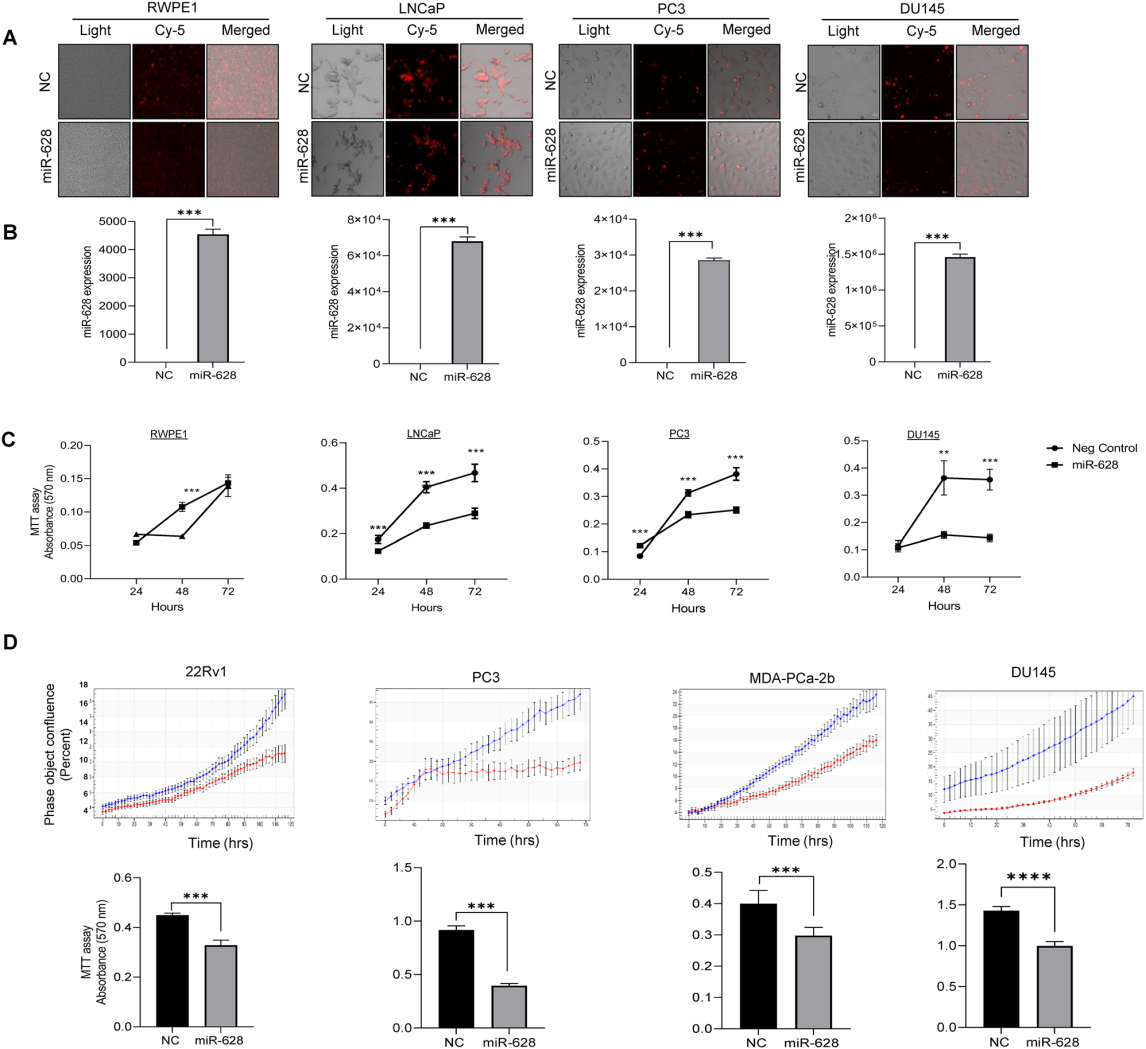
Overexpression of miR-628 affects PCa cell proliferation. (**A**) Cy-5 labeled miR-628 mimic or NC (100 nM) was transiently expressed in RWPE-1 and PCa cells. Effective transfection was visualized using immunofluorescence (red). Images were captured under Zeiss confocal microscope and representative images are shown at 20 × magnification. (**B**) Transfection efficiency of both miR-628 and NC was confirmed through RT/qPCR (***p < 0.001). (**C**) The effects of overexpression of miR-628-5p expression on cell proliferation were assessed through MTT assay in RWPE-1 cells or in PCa cells transfected with 100 nM of miR-628 mimic or NC for 24, 48, and 72 h (**p < 0.01, ***p < 0.001). (**D**) Live cell analysis was performed by using Incucyte in PCa cells transfected with 100 nM of miR-628-5p mimic (red line) or NC (blue line) for 72 or 120 h (upper panels). Results were further confirmed with MTT assay (lower panels). At the end of the selected period, absorbance was read at 570 nm (***p < 0.001). Data represent average ± SEM. Experiments were performed in triplicates.

Since the expression of miR-628 is downregulated by leptin in PCa tumors and PCa cells, we asked whether altering miR-628 expression affects PCa cell survival and proliferation. For this purpose, we transfected four PCa cell lines with CY labeled miR-628 mimic or Negative Control (NC) mimics. The resulting expression levels were assessed using a fluorescence microscope to determine relative transfection efficiency (Fig. 3A). We confirmed efficient transfection of both miR-628 mimic and NC in all four cell lines (Fig. 3A). MiR-628 expression was significantly increased after transfection of miR-628 mimic, as shown by RT/qPCR (Fig. 3B).

Further, the effect of miR-628 on cell survival was analyzed by MTT assay on indicated prostate RWPE1 cells and PCa cell lines. We observed a modest reduction in cell survival in RWPE1 cells at 48 h but not significant at the 72 h time point (Fig. 3C, left panel). When we analyzed the impact of miR-628 on cell survival in the PCa cell lines (PC3, LNCaP, and DU145), we found that overexpression of miR-628 significantly reduced cell survival in all indicated PCa cell lines (**p < 0.01, ***p < 0.001) (Fig. 3C). Interestingly, PC3 cells were minimally responsive at earlier time points (Fig. 3C) but were significantly affected at later time points.

We next investigated the effect of miR-628 on PCa cell proliferation using live-cell analysis by Incucyte for 72 or 120 h (Fig. 3D, upper panels), and the results were further confirmed with MTT assay at the end of the selected period (Fig. 3D, lower panel). Our data demonstrated that expression of miR-628 mimic (red line) effectively impaired cell proliferation in all the PCa cell lines over time compared to NC control (blue line), and the reduction in cell proliferation was statistically significant when measured by MTT assay (Fig. 3D, lower panel). Collectively our data indicate that miR-628 overexpression reduced cell survival/proliferation in PCa cell lines.

### Expression of miR-628 affects cell colony and spheroid formation in PCa cells

**Figure 4:**
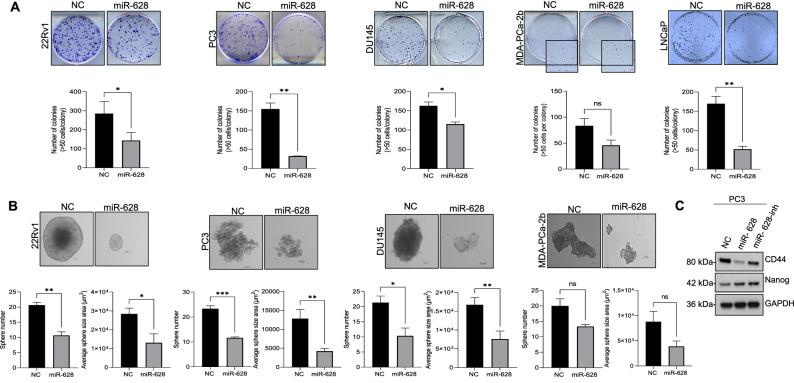
Expression of miR-628 affects cell colony and spheroid formation in PCa cells. (**A**) PCa cells were transfected with 100 nM of miR-628 or NC and cell colony formation assay was performed (upper panels) as described in the materials and methods section and the number of colonies was quantified using ImageJ and plotted using GraphPad PRISM (lower panels) *p < 0.05, **p < 0.01. (**B**) Spheroid formation assay was performed in PCa cell lines after transfected with 100 nM of miR-628 or NC as described in the materials and methods section. Images were captured using an inverted light microscope and representative images are shown at ×20 magnification (upper panels). The effect of miR-628 on spheroid number and sphere size was quantified using the Zeiss ZEN Blue lite software (lower panels) and plotted using GraphPad PRISM. Data represent average ± SEM. *p<0.05, **p< 0.01, ***<0.001. (**C**) Protein expression of CD44 and Nanog is shown in PC3 cells after transfection with 100 nM of miR-628 mimic, miR-628 inhibitor, or NC. Full-length images are provided in supplementary information files.

To further characterize the effects of miR-628 overexpression in PCa, we performed a cell colony formation assay after overexpression of miR-628 or NC. Cell colonies were defined with at least 50 cells/colony 10 days after plating (Fig. 4A). We selected PC3, DU145, and LNCaP PCa cells as in previous experiments and added another PCa cell line MDA-PCa-2b. Transfection with miR-628 mimic significantly reduced colony formation in 22Rv1 (50% reduction), PC3 (79% reduction), DU145 (45% reduction), MDA-PCa-2b cells (37% reduction) and LNCaP cells (69% reduction) compared to cells transfected with NC mimic (*p < 0.05, **p < 0.01, ns-not significant) (Fig. 4A, upper and lower panels). It is known that the ability of cells to form spheres is an indicator of the percentage of cells with stem-like characteristics present in a population of cells^26^. To investigate the role of miR-628 in this hallmark of cancer development, we transfected PC3, 22Rv1, MDA-PCa-2b, and DU145 cells with NC control or miR-628 mimic, and cells were grown in non-adherent plates under serum-free conditions, and spheroid development was observed after 10 days (Fig. 4B, upper panel). A significant reduction in both spheroid size and the number was observed after transfection with miR-628 mimic compared to cells transfected with NC in the PCa cell lines (22Rv1, PC3, MDA-PCa-2b, and DU145) (*p<0.05, **p<0.01, ***p<0.001, ns-not significant) (Fig. 4B, upper and lower panels).

Furthermore, we studied the expression of CD44 and Nanog, commonly used cancer stem-cell markers^27^ after transfection of PC3 cells with NC, miR-628, and miR-628 inhibitor. We observed that PC3 cells transfected with miR-628 mimic had decreased expression of CD44 compared to cells transfected with NC mimic control (Fig. 4C). However, this effect was not observed when the expression of Nanog was evaluated. Interestingly, transfection with miR-628 inhibitor (100 nM) had the opposite effect, increasing the expression of Nanog compared to mimic. The expression of CD44 also increased when PC3 cells were transfected with miR-628 inhibitor compared to cells transfected with miR-628 mimic (Fig. 4C). Together, these results indicate that miR-628 affects PCa cell proliferation and reduces cell colony formation and spheroid formation in PCa cells. These data support the potential role of miR-628 in regulating properties related to PCa cell stemness.

### MiR-628 targets *JAG1*-3′UTR and affects JAG1 expression and downstream Notch signaling PCa cells

**Figure 5:**
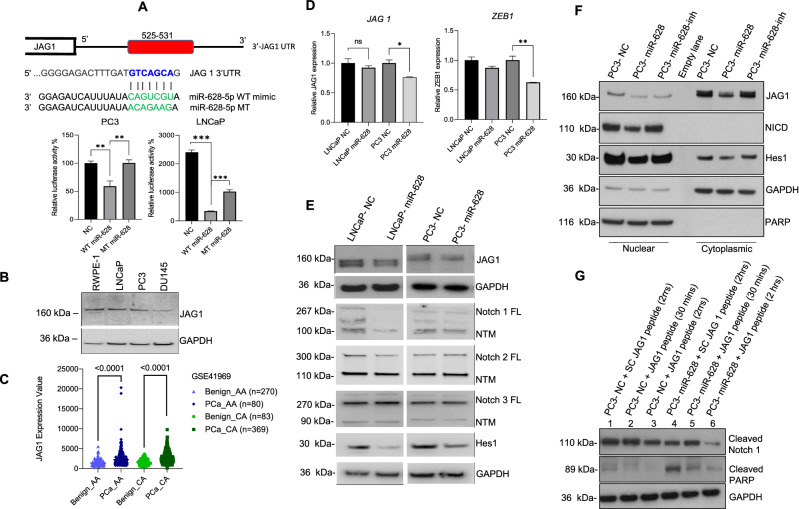
MiR-628 targets JAG1 in PCa cells and affect JAG1 downstream Notch signaling in PCa cells. (**A**) Jagged-1 was identified as a potential miR-628 target using Targetscan. Wild type and mutant target sequences of miR-628 are shown. miR-628 and *JAG1*- 3′UTR interaction was assessed through luciferase assay in LNCaP and PC3 cells transfected with wild type miR-628 (WT) or mutant type miR-628 (MT) (**p < 0.01, ***p < 0.001). (**B**) *JAG1* expression was assessed in RPWE-1 and PCa cells by western blotting. Membranes were cut before hybridization using the molecular weight marker as a reference and imaged using an Azure imaging system. All western blot full images are provided in supplementary information files. (**C**) JAG1 expression was assessed utilizing the GSE41969 dataset and analyzed using the GEO2R web tool in matched benign and PCa samples obtained from AA and CA men. Light blue triangles = benign AA (n = 80), dark blue diamonds = PCa AA (n = 270), light green circles = benign CA (n = 83), and dark green squares = PCa CA (n = 369), (p < 0.0001). (**D**) Effect of mir-628 on the expression of *JAG1* and *ZEB1* expression as measured by RT/qPCR in LNCaP and PC3 cells after transfection with 100 nM of miR-628 mimic or NC (*p < 0.05, **p < 0.01). (**E**) Protein expression of JAG1, Notch 1, Notch 2, Notch 2, and Hes1 is shown in LNCaP and PC3 cells after transfection with 100 nM of miR-628 mimic or NC. Membranes were cut before hybridization using the molecular weight marker as a reference and imaged using an Azure imaging system. All western blot full images are provided in supplementary information files. (**F**) Protein expression of JAG1, Notch intracellular domain (NICD), and Hes1 are shown in PC3 cells nuclear and cytosolic fractions after transfection with 100 nM of miR-628 mimic, miR-628 inhibitor, or NC. PARP and GAPDH were used as loading controls for nuclear and cytosolic protein fractions and imaged using film. (**G**) Protein expression of cleaved Notch1, cleaved-PARP are shown in PC3 cells after transfection with 100 nM of miR-628 mimic, miR-628 inhibitor, or NC and treatment with Scrambled control JAG1-peptide (SC) or JAG1-peptide for 30 min or 2 h and imaged using film.

To determine the mechanistic role of miR-628 in PCa suppression we used the Target scan software (http://www.targetscan.org/vert_72/) to analyze genes possibly targeted by miR-628. Target scan analysis suggests that miR-628 can bind with several targets, including the 3′UTR of *JAG1*, a ligand of Notch signaling (Fig. 5A, upper panel). JAG1 overexpression has been correlated with PCa disease recurrence, therapy resistance, and metastasis^28,29^. To confirm this, we performed luciferase assays to determine if miR-628 can activate or repress the expression of *JAG1*. Co-transfection of *JAG1*-3′UTR-Luc construct with wild-type miR-628 decreased luciferase activity; however, mutant type miR-628 expression restored luciferase activity significantly in both LNCaP and PC3 cells (Fig. 5A, lower panel). We also assessed JAG1 expression in RWPE-1 and various PCa cell lines. Immunoblotting data shows that the expression of JAG1 protein is variable between RWPE1 and PCa LNCaP, PC3, and DU145 cells (Fig. 5B). Furthermore, to study if *JAG1* was differentially expressed between “matched normal” or benign and PCa samples obtained from AA and CA men, we utilized dataset GSE41969 from the GEO and analyzed it using the GEO2R web tool. We had 163 benign and 629 PCa samples (80 benign AA samples, 270 PCa AA samples, 82 benign CA samples, and 369 CA samples). *JAG1* was significantly overexpressed in both AA and CA PCa samples compared to their respective normal matched controls (p < 0.0001) (Fig. 5C). *JAG1* was also statistically overexpressed in the tumor samples compared to the benign samples, regardless of race (p < 0.0001) (Fig. 2S).

We chose PCa cell lines LNCaP and PC3 to continue future studies. We quantified the mRNA levels of *JAG1* after transfection of miR-628, and our data indicate that overexpression of miR-628 downregulated mRNA levels of *JAG1* and its downstream target *ZEB1* significantly in PC3 cells (Fig. 5D, left and right panels). An earlier study suggested that as a Notch receptor ligand, JAG1 promotes epithelial-mesenchymal transition (EMT)^30^. Since miR-628 affects *JAG1* expression, we analyzed the effect of miR-628 on the expression of JAG1, and its receptors Notch1, Notch2, Notch3, and Notch downstream target Hes1. Immunoblotting data demonstrated that miR-628 overexpression downregulated the expression of Notch ligand, JAG1, and also downregulated the expression of Notch1, Notch2, Notch3, and Notch downstream marker HES1 in both LNCaP and PC3 cell lines (Fig. 5E) suggesting that miR-628 targets JAG1 and inhibits Notch signaling in PCa cells.

Further, we examined the effect of miR-628 overexpression or knockdown on JAG1 cytosolic and nuclear shuttling. We observed a decrease in JAG1 in both nuclear and cytosolic fractions compared to NC controls. JAG1 expression increased when PC3 cells were transfected with miR-628 inhibitor. The expression of the Notch intracellular domain (cleaved notch) revealed that overexpression of miR-628 decreased detection of NICD in the nuclear fraction and inhibitor transfection had opposite effects. Finally, we observed a decrease in downstream target Hes1 in both nuclear and cytosolic fractions of PC3 cells transfected with miR-628. On the other hand, transfection with miR-628 inhibitor increased Hes1 expression compared to miR-628 mimic (Fig. 5F). Together, these results suggest that miR-628 targets JAG1 and its downstream signaling in PCa cells.

To determine if activation of the JAG1-Notch pathway is affected by miR-628 expression, we utilized a JAG1-mimicking peptide to initiate activation of this pathway through the measurement of Notch1 cleavage (Fig. 5G). Similar JAG1 peptides have been previously utilized to study the effects of the activation of this pathway under a variety of conditions^31^. We first treated PC3 cells with 100 nM of miR-628 mimic or NC for 48 h and then initiated the JAG1-Notch pathway by treating the cells with 100 µM of JAG1-peptide or a Scramble JAG1-peptide (SC) as control. We observed an increase in cleaved Notch1 expression in PC3 cells treated with NC for 30 min. after initiation of the Jagged-Notch pathway compared to SC. Interestingly, Notch1 cleavage was reduced in PC3 cells transfected with miR-628 compared with PC3 cells treated with NC under the same conditions. We did observe an increase in cleavage 30 min after initiation of the Jagged-Notch pathway using JAG1 peptide compared to SC, with a decrease at 2 h. Next, we assessed PARP cleavage under these same conditions. We observed minimal cleavage in PC3 cells transfected with NC control and treated with either SC or JAG1 peptide. On the other hand, we observed an increase in PARP cleave in PC3 cells transfected with miR 628 and treated with SC. The observed PARP cleavage was reduced after initiation of the JAG1-Notch pathway using the JAG1 peptide, showing a possible override of the effects of miR 628 mimic. Together, these results suggest that miR-628 targets JAG1 and may modulate the expression of its targets.

### MiR-628 expression affects PCa cell migration, invasion, and tube formation in HUVEC cells

**Figure 6:**
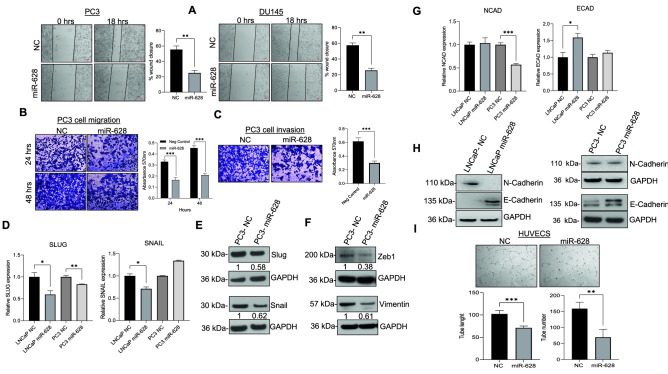
MiR-628 expression affects PCa cell migration, invasion, and tube formation in PCa and HUVEC cells. (**A**) Cell motility assay (scratch assay) was performed in PC3 and DU145 cells transfected with miR-628 (100 nM) or NC (100 nM) (representative images, left panels) and scratch closure was quantified after 18 h using ImageJ (right panels; **p < 0.01). (**B**) Transwell cell migration was assessed in PC3 cells transfected with miR-628 or NC. Representative images are shown in upper panels taken at 10X magnification with a light microscope. Absorbance (562 nm) was determined at 24 and 48 h (lower panels) as described in the material and method section. ***p < 0.001. (**C**) Cell invasion assay assessed in PC3 cells transfected with miR-628 (100 nM) or NC (100 nM) (upper panels) and absorbance (562 nm) was determined at 48 h (lower panels; ***p < 0.001). Images were taken at a 10X magnification with a light microscope. (**D**) The effect of miR-628 on the expression of *SLUG* and *SNAIL* expression was measured by RT/qPCR in LNCaP and PC3 cells after transfection with 100 nM of miR-628 mimic or NC. *p < 0.05, **p < 0.01. (**E**) Protein expression of Slug and Snail are shown in PC3 cells after transfection with 100 nM of miR-628 mimic or NC and imaged using film. (**F**) Protein expression of Zeb1 and Vimentin are shown in PC3 cells after transfection with 100 nM of miR-628 mimic or NC and imaged using film. (**G**) The effect of miR-628 on the expression of E-cadherin (*ECAD)* and N-cadherin (*NCAD)* was measured by RT/qPCR in LNCaP and PC3 cells after transfection with 100 nM of miR-628 mimic or NC (left panels). *p < 0.05, ***p < 0.001 (**H**) E-cadherin and N-cadherin protein expression was analyzed by Western blotting as described in the materials and methods section after transfection with 100 nM of miR-628 mimic or NC. Membranes were cut before hybridization using the molecular weight marker as a reference and imaged using an Azure imaging system. All western blot full images are provided in supplementary information files. (**I**) Angiogenesis was determined in HUVEC cells transfected with miR-628 or NC (upper panels). Representative images were captured using an inverted light microscope and representative images are shown at 20 × magnification. The tube length and number were determined at 24 h using ImageJ (lower panels; **p < 0.01, ***p < 0.001).

Since miR-629 targets the 3′UTR region of *JAG-1*, we wanted to study the effect of miR-628 overexpression in different downstream targets and PCa cell migration, invasion, and its impact on tube formation in HUVEC cells. Using an in vitro scratch assay, we quantified the effect of miR-628 on PCa cell migration (Fig. 6A). Our data suggest that overexpression of miR-628 significantly reduced cell migration in PC3 (48%) and DU145 (55%) cells compared with NC transfected PC3 (28%) and DU145 (29%) cells (Fig. 6A, left and right panels). The effect of miR-628 on PCa cell migration and invasion was further analyzed by trans-well assay (Fig. 6B,C). MiR-628 overexpression downregulated PC3 cell migration compared to NC mimic control (49% after 24 h and 45% after 48 h) (Fig. 6B) and decreased invasion in PC3 cells transfected with miR-628 (48% after 48 h) compared with NC transfected PC3 cells (Fig. 6C). Mechanistically the expression of Snail/Slug and EMT markers play an important role in cell migration, invasion, and aggressiveness in PCa tumor cells^32,33^. RT/qPCR data indicate that overexpression of miR-628 inhibits Snail and Slug expression in LNCaP cells and Snail expression in PC3 cells (Fig. 6D). We also assessed Snail and Slug protein expression via immunoblotting in PC3 cells transfected with miR-628 mimic or NC (Fig. 6E), and our data suggest that PC3 cells transfected with miR-628 showed a decreased expression of Slug (0.58) and Snail (0.61) compared to NC (Fig. 6E). PC3 cells transfected with miR-628 had decreased expression of zinc finger E-box-binding homeobox 1 (ZEB1) (0.38) and Vimentin (0.61) compared to cells transfected with NC control (normalized to 1 for each protein) (Fig. 6F). Increased expression of ZEB1 has been shown to enhance motility and invasiveness, thus promoting an aggressive phenotype in PCa cells^34^. We also analyzed the expression of EMT markers E-cadherin and N-cadherin after overexpression of miR-628 by RT/qPCR (Fig. 6G) and immunoblotting (Fig. 6H). Overexpression of miR-628 decreased expression of N-cadherin mRNA levels in PC3 cells, but no significant changes were observed at the protein level. Transfection of miR-628 led to decreased protein expression of N-cadherin in LNCaP cells, but no significant changes were observed when mRNA was measured. Overexpression of miR-628 decreased expression at both the mRNA and protein levels of E-cadherin in LNCaP cells and decreased its protein expression in PC3 cells (Fig. 6G,H). Since effective tumor growth and eventual metastasis are dependent on angiogenesis^35^, we next investigated the role of miR-628 in angiogenesis (Fig. 6I). HUVEC cells were transfected with miR-628 mimic or NC mimic for 24 h, cells were re-plated on matrigel coated plates, and vascular tube formation was monitored after 24 h (Fig. 6I). Our results show that miR-628 significantly reduced tube number (**p < 0.01) and length (pixels) (***p < 0.001) compared to HUVEC cells transfected with NC. These results show that miR-628 expression downregulates PCa cell hallmarks, namely migration, invasion, and angiogenesis.

### Expression of miR-628 increases sensitivity towards enzalutamide and docetaxel and induces apoptosis

Drug resistance is one of the main drivers of adverse outcomes in PCa patients^36^. Drugs such as enzalutamide (ENZ) effectively inhibit androgen receptor (AR) signaling, which plays an important role in the development of PCa disease^37^. Docetaxel (DTX) binds tubulin and stabilizes microtubules, improving overall survival in men suffering from metastatic disease^38^. Since the aberrant expression of miRNAs has been linked to chemoresistance and the development of CRPC^39,40^, we explore the role of miR-628 in drug resistance.

**Figure 7:**
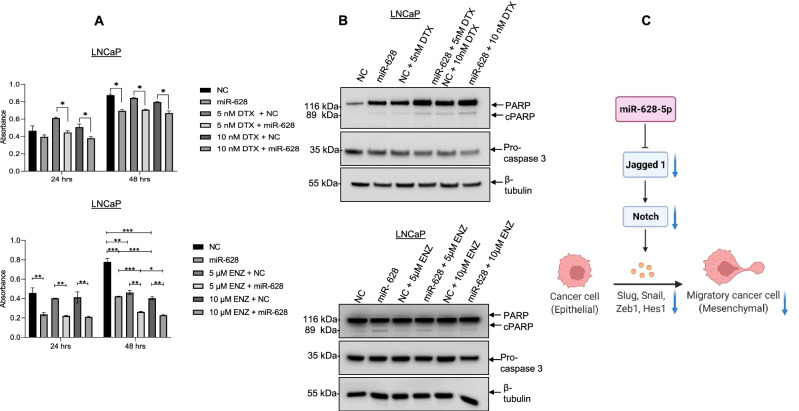
Overexpression of miR-628 and treatment of Enzalutamide or DTX decreases cell proliferation and induces apoptosis in LNCaP. (**A**) LNCaP cells transfected with miR-628 mimic or NC were treated with enzalutamide (ENZ) (5 µM, or 10 µM) or docetaxel (DTX) (5 nM or 10 nM). Cell viability was measured after 24 h and 48 h after transfection using MTT assay (*p < 0.05, **p < 0.01, ***p < 0.001). (**B**) The effect of miR-628 and co-treatment of ENZ or DTX on PCa cell apoptosis was assessed through cleaved-PARP and pro-Caspase-3 expression using western blotting as described in the materials and methods section. Membranes were cut before hybridization using the molecular weight marker as a reference and imaged using an Azure imaging system. All western blot full images are provided in supplementary information files. (**C**) Schematic model representing the anti-PCa role of miR-628. MiR-628 targets Jagged1 affecting Notch signaling and EMT.

Both LNCaP (AR-positive) and PC3 (AR-negative) cells were transfected with NC or miR-628, and cells were then treated with increasing concentrations of Enz (5 µM and 10 µM) or DTX (5 nM and 10 nM), and relative cell survival was measured by MTT assay (Fig. 7A, Fig. 3S). Expression of miR-628 decreased cell survival after 48 h compared with NC transfected cells as expected (Fig. 7A, Fig. 3SA upper and lower panels). LNCaP cells transfected with miR-628 did not show a significant decrease in cell survival when treated with increasing concentrations of DTX as compared to NC transfected and vehicle-treated cells (Fig. 7A, upper panels). In contrast, PC3 cells transfected with miR-628 did show a significant decrease in cell survival after treatment with increasing concentrations of DTX compared with NC transfected and vehicle-treated PC3 cells (Fig. 3SA, lower panels). Moreover, miR-628 overexpression and ENZ treatments significantly decreased LNCaP cell survival compared to miR-628 transfected cells, and no significant ENZ effects were observed in PC3 cells since PC3 cells do not express androgen receptor (AR). In addition, we analyzed the effect of miR-628 on cell apoptosis after treatment of DTX and ENZ. Our data suggested that overexpression of miR-628 induced cleaved-PARP expression and decreased pro-Caspase-3 expression, indicating that miR-628 induced cell apoptosis in both LNCaP and PC3 cells (Fig. 7B, Fig. 3SB, upper and lower panels). Interestingly, overexpression of miR-628 combined with DTX or ENZ treatments also induced cleaved-PARP expression and decreased pro-Caspase-3 expression in both LNCaP and PC3 miR-628 cells compared with NC and DTX or ENZ treated cells (Fig. 7B, Fig. 3SB, upper and lower panels). These results suggest that a combination of miR-628 and the clinically approved drugs ENZ and DTX could have synergistic or additive effects in decreasing PCa cell proliferation and possibly enhancing cell death.

## Discussion

Obesity is a risk factor for both cancer development and increased mortality from this disease^41^. Due to the ubiquitous nature of adipose tissue, many types of solid tumors grow close to or in direct contact with adipocytes and other adipose-associated cell populations. The specific nature of the reciprocal communication between a developing tumor and adjacent adipose tissue is an area of active study. A growing body of literature indicates that these interactions with the local adipose milieu are important drivers of malignancy^42^. Adipocytes exhibit both short- and long-range interactions with cancer cells and can regulate gene expression in other peripheral tissues through the secretion of adipokines. For example, blood levels of adipokines are associated with PCa development^9^. The complicated crosstalk between obese-related adipokines, such as leptin, includes post-transcriptional gene regulation that may result in an altered miRNA transcriptome that may affect tumor proliferation, invasion, apoptosis, and angiogenesis^17^. Overall, our results show that increased leptin levels can result in a significant decrease in miR-628 expression and that this decrease increases markers of PCa cell aggressiveness. This has serious implications since obesity has already been associated with the risk of developing aggressive disease and recurrence due to decreased biopsy and prostate-specific antigen (PSA) efficiency and overall larger prostate size^43^. Regulation of miRNAs by hormones such as leptin could further explain the complex interaction of adipose and surrounding tissue that contributes to PCa disease, especially in cases where there is a high level of adiposity and adipokine secretion. Our results show that co-treatment with miR-628 and leptin partially abrogated the proliferative effects of this adipokine. A more comprehensive understanding of the role of leptin in regulating miRNAs in PCa is necessary, and the novelty of this discovery could potentially lead to more insights into the development of this disease.

MiRNAs are small non-coding RNA molecules with specific sequences that bind to corresponding target sequences in mRNA or DNA and suppress gene expression^44^. Aberrant expression of miRNAs has been shown to contribute to a wide range of diseases, including cancer^44^. In the current study, we found that miR-628 expression levels were lower in the PCa cell lines compared to non-cancer-derived prostate cells. This is consistent with our previous study^20^ showing that patients with PCa have decreased expression of miR-628 compared to controls. In addition, increasing expression or “re-expression” of miR-628 effectively repressed tumorigenic properties in PCa cell lines, namely proliferation, colony formation, migration, and invasion. These results suggest that the decrease or loss in miR-628 expression might play a role in the development of PCa. Interestingly, increased expression of miR-628 in HUVEC cells decreased tube size and number compared to control, effectively abrogating angiogenesis. Increased angiogenesis and lymphangiogenesis are hallmarks of cancer formation, aiding cells in surviving the hypoxic conditions associated with solid tumors^45^. This is a relatively unexplored aspect of miR-628 function and warrants further study.

MiR-628 can target multiple genes, but we chose to explore its interaction with Jagged-1 due to its relevance in PCa and its correlation with aggressive PCa and poor prognosis^28^. MiR-628 directly targets the 3′UTR of *JAG1* and decreases the expression of *JAG1* at both the mRNA and protein levels. Furthermore, our luciferase gene reporter assay showed that mutations in the interacting region of miR-628 effectively upregulated the *JAG1-*Luc activity, confirming the binding of miR-628 to the UTR of *JAG1*. Next, we observed that miR-628 overexpression in PCa cell lines effectively decreased levels of multiple *JAG1* downstream targets. JAG1 is one of five canonical ligands of Notch receptors, and the ligand-induced Notch signaling system is a well-characterized signaling system that plays critical roles in development, tissue homeostasis, and disease^29^. Dysregulation of Notch signaling has been found in various diseases, including cancer, and several studies reported the involvement of JAG1 in cancer cell invasiveness, EMT, and metastasis^46^. These results highlight that miR-628 re-expression could potentially abrogate the activation of the JAG1-Notch pathway and decrease EMT, which is essential for tumorigenesis. Notably, the variation in mRNA and protein levels observed in some of the targets could be attributed to various factors, such as post-transcriptional and post-translation regulation and cellular response to highly dynamic scenarios, particularly under stressful conditions, such as a cancer cell microenvironment^46–49^. We also observed that Nanog levels were not affected by the miR-628 mimic compared to NC control, but a robust overexpression was observed when the miR-628 inhibitor was utilized. It seems that miR-628 effects in Nanog specifically are not as significant when overexpressed but rather when the inhibitor is utilized to decrease miR-628 expression. Although we do not have evidence that miR-628 directly binds to the 3′UTR region of Nanog, we can hypothesize that absence of miR-628 might contribute to changes in the expression of another miR-628 target that regulates Nanog or could be the effects of post-transcriptional modifications.

In our studies, we wanted to see the effects of combining miR-628 mimic and clinically relevant drugs such as ENZ and DTX to study if co-treatment could increase the effectiveness of these drugs. Although PC3 cells are AR-independent cell lines and the effects of ENZ treatment in proliferation were not as significant, we did observe an increase in PARP cleave and a reduction in the pro-caspase 3. The PC3 cells could be utilized to study AR-independent disease and the ENZ resistance model since cancer cells can develop alternative mechanisms, such as GR overexpression, to effectively develop therapeutic strategies to circumvent the effects of AR blockage^50^.

Due to their stability in various body fluids such as blood and serum, miRNAs are used as diagnostic tumor-specific biomarkers in cancer^51^. Our group previously reported a significant decrease in miR-628 in the plasma of PCa patients compared to controls, thus identifying MiR-628 as a promising marker for this disease^20^. This is significant given that the currently utilized biomarker for PCa, PSA, has limited diagnostic usefulness due to the variability in expression in both serum and cancer tissue^52^. This biomarker variability could result in unnecessary biopsy and even removal of the prostate in cases where the disease was not likely to become aggressive^53^. On the other hand, revised recommendations for delayed testing have left the AA males vulnerable to a more aggressive disease with higher mortality in their community and a higher tumor volume despite similar PSA levels to their Caucasian counterparts^54^. This highlights the importance of utilizing additional markers of disease, such as miR-628 to appropriately identify and screen men that could potentially develop more aggressive PCa disease.

Overall, our results show that miR-628 is downregulated in PCa patient tissue, and its overexpression reduced key cancer hallmarks, including proliferation, colony formation, invasion, migration, angiogenesis, and decreased drug resistance. We also show that miR 628 directly targets *JAG1* transcription and its overexpression downregulated specific downstream targets in the JAG1-NOTCH signaling pathway involved in EMT, as presented in our model (Fig. 7C). Furthermore, leptin downregulated miR-628 and increased cell proliferation, while overexpression of miR-628 could partially abrogate the proliferative effects of this hormone. Overall, miR-628 effects in PCa cells have significant implications for PCa development, and together with our previous work, miR-628 could be further explored as a promising biomarker for the disease.

## Supplementary Information

Supplementary Information 1.

Supplementary Information 2.

Supplementary Information 3.

Supplementary Information 4.

Supplementary Information 5.

Supplementary Information 6.

## Data Availability

All data generated during the current study are included in this article and will be available from the corresponding author on request.
