# Supplementary material for: Leptin modulated microRNA-628-5p targets Jagged-1 and inhibits prostate cancer hallmarks
Source: Sci Rep. 2022 Jun 16;12:10073. doi: 10.1038/s41598-022-13279-x (PMC9203512; doi:10.1038/s41598-022-13279-x)

Figure 2S

Leptin modulated microRNA-628-5p targets Jagged-1 and inhibits prostate cancer hallmarks

Leslimar Rios-Colon[1,2*], Juliet Chijioke[1*], Suryakant Niture[1], Zainab Afzal[1], Qi Qi[1], Anvesha Srivastava[1], Malathi Ramalinga[1], Habib Kedir[1], Patrice Cagle[1], Elena Arthur[1], Mitu Sharma[2], John Moore[1], Gagan Deep[2,3,4] Simeng Suy[5], Sean P Collins[5], and Deepak Kumar[1**]

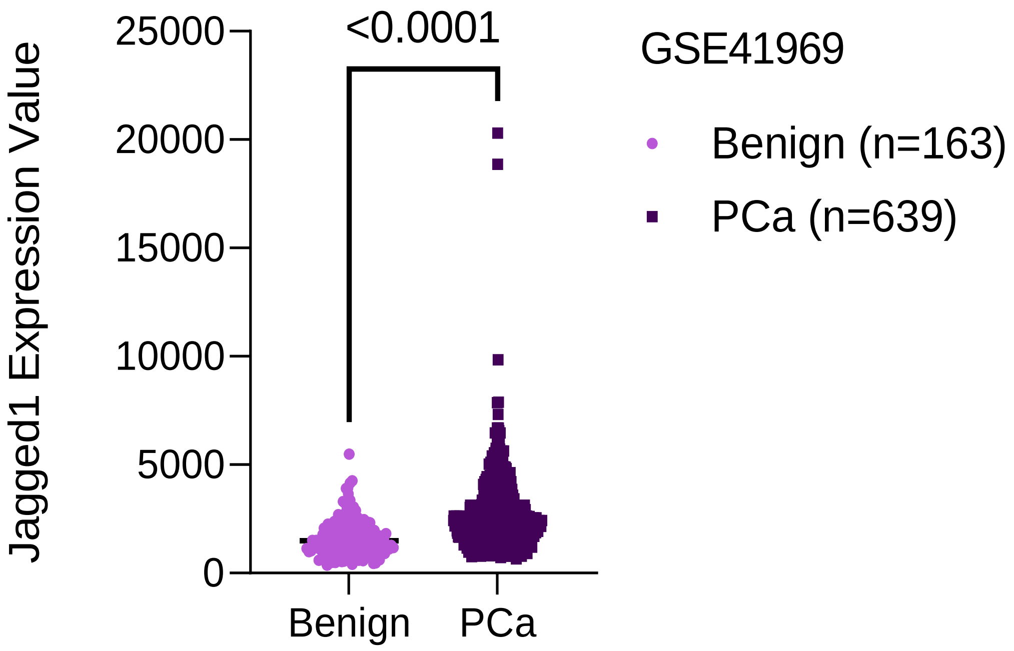

Supplement: Supplementary file 2 — Supplementary Information 2. [file 41598_2022_13279_MOESM2_ESM.pdf]
