# Supplementary material for: Leptin modulated microRNA-628-5p targets Jagged-1 and inhibits prostate cancer hallmarks
Source: Sci Rep. 2022 Jun 16;12:10073. doi: 10.1038/s41598-022-13279-x (PMC9203512; doi:10.1038/s41598-022-13279-x)
Supplement: Supplementary file 4 — Supplementary Information 4. [file 41598_2022_13279_MOESM4_ESM.pdf]

Leptin modulated microRNA-628-5p targets Jagged-1 and inhibits prostate cancer hallmarks

Leslimar Rios-Colon[1,2*], Juliet Chijioke[1*], Suryakant Niture[1], Zainab Afzal[1], Qi Qi[1], Anvesha Srivastava[1], Malathi Ramalinga[1], Habib Kedir[1], Patrice Cagle[1], Elena Arthur[1], Mitu Sharma[2], John Moore[1], Gagan Deep[2,3,4] Simeng Suy[5], Sean P Collins[5], and Deepak Kumar[1**]

[1]Julius L. Chambers Biomedical Biotechnology Research Institute, North Carolina Central University, Durham, North Carolina NC; [2]Wake Forest University Health Sciences, Winston-Salem, NC, [3]Wake Forest Baptist Comprehensive Cancer Center, North Carolina; [4]Department of Urology, Wake Forest School of Medicine, Winston-Salem, North Carolina, [5]Lombardi Comprehensive Cancer Center, Georgetown University, Washington

Supplementary materials
Western blots

Figure 4

C

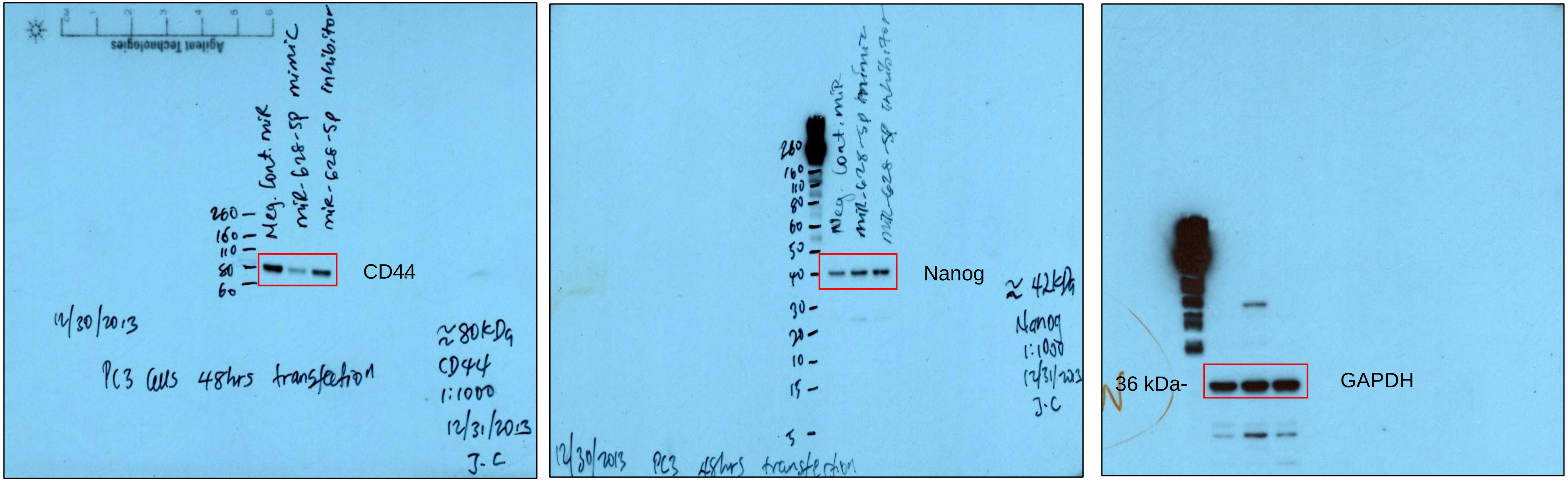

Full length images of blots for Fig. 4C in main paper. Red rectangles indicate the regions used in the figures. PVDF membranes were used for probing with the respective primary antibody. Film was utilized to develop membranes.

Figure 5

B

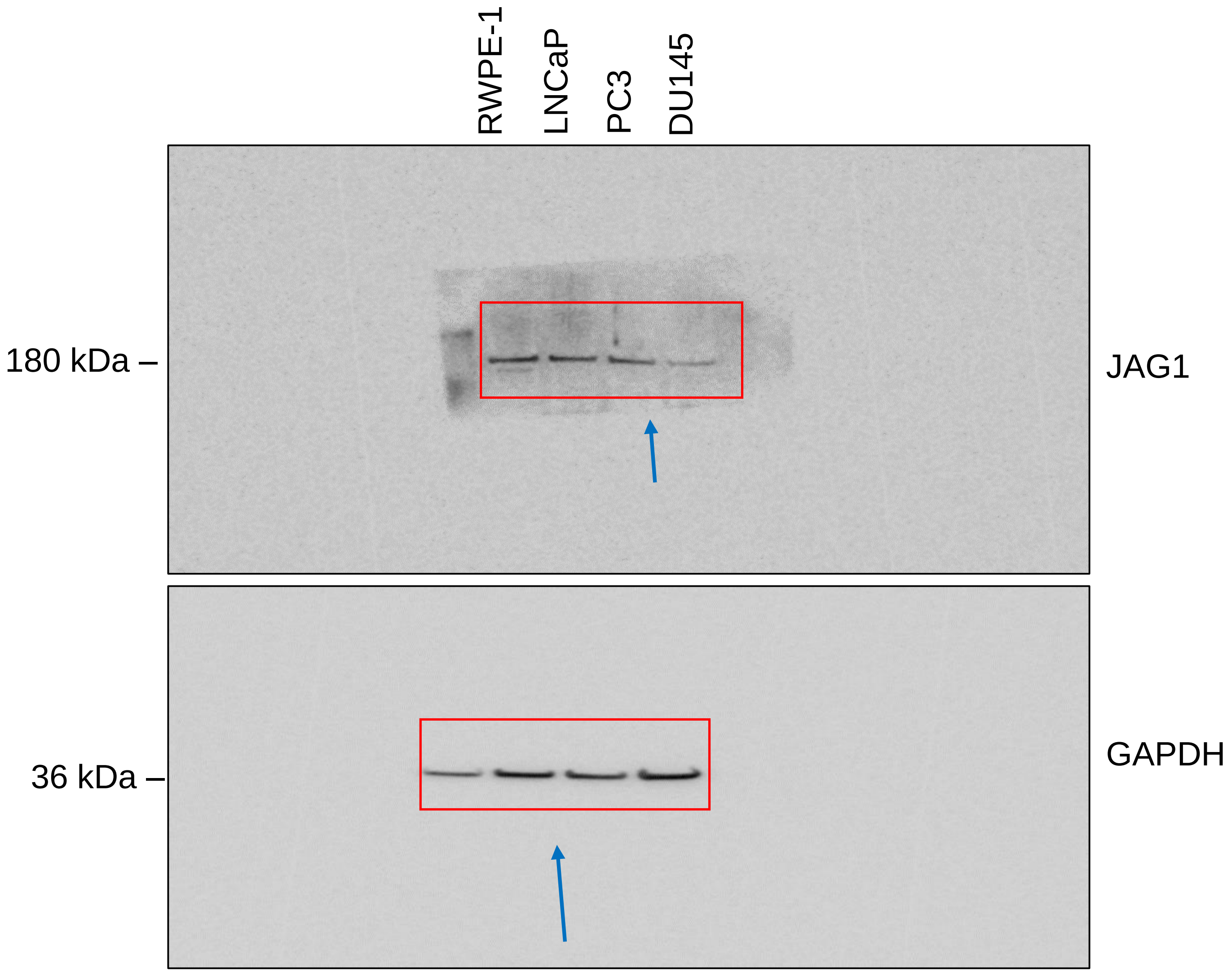

** membrane was cut previous to probing using molecular weight marker as reference. Edges are marked with blue arrow when possible. The Azure c500 was used to scan membranes.

Full length images of blots for Fig. 5B in main paper. Red rectangles indicate the regions used in the figures. PVDF membranes were cut where indicated using molecular weight marker as reference, and used for probing with the respective primary antibody.

Figure 5

E

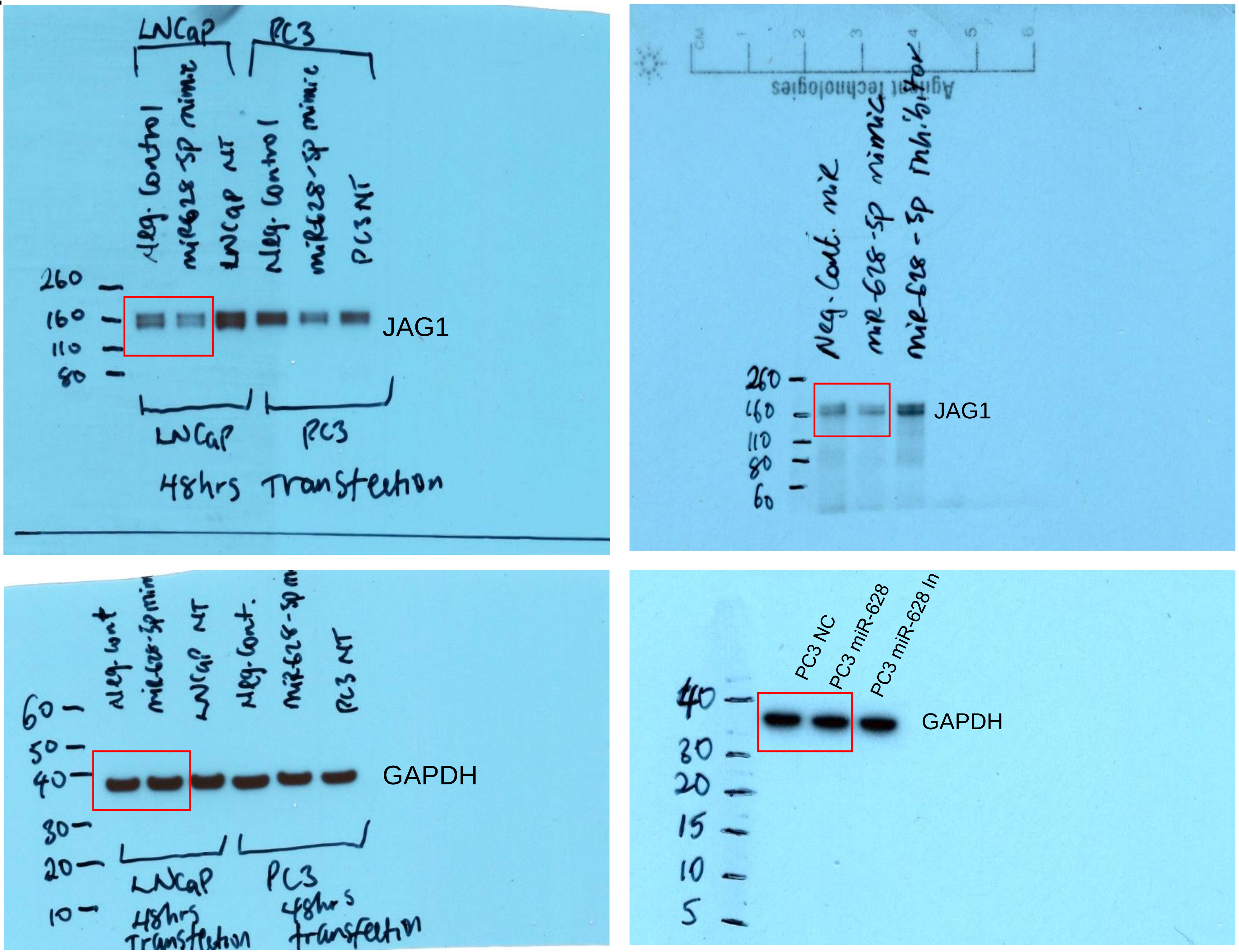

Full length images of blots for Fig. 5E in main paper. Red rectangles indicate the regions used in the figures. PVDF membranes were used for probing with the respective primary antibody. Film was utilized to develop membranes.

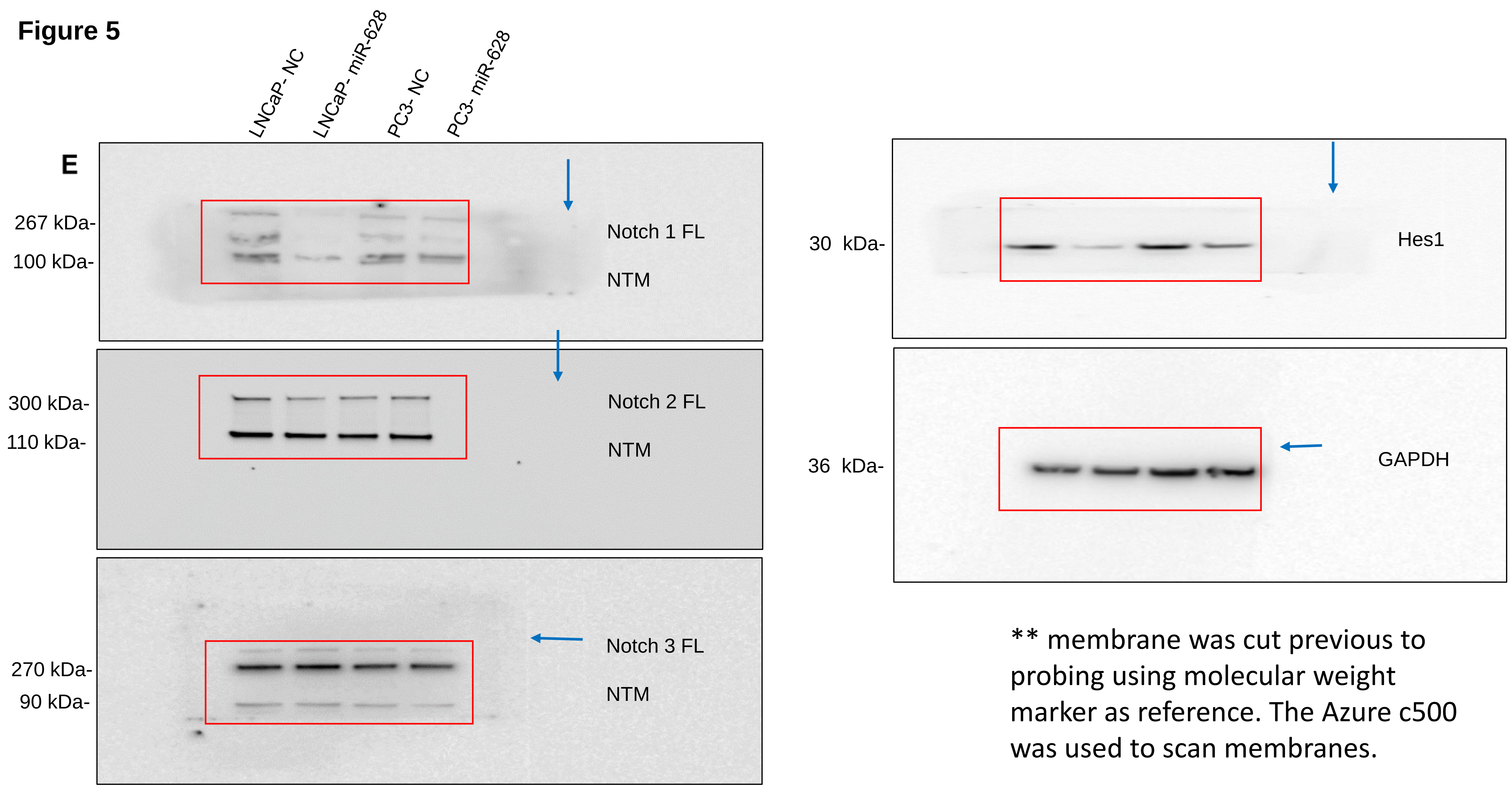

** membrane was cut previous to probing using molecular weight marker as reference. The Azure c500 was used to scan membranes.

Full length images of blots for Fig. 5E in main paper. Red rectangles indicate the regions used in the figures. PVDF membranes were used for probing with the respective primary antibody.

Figure 5

F

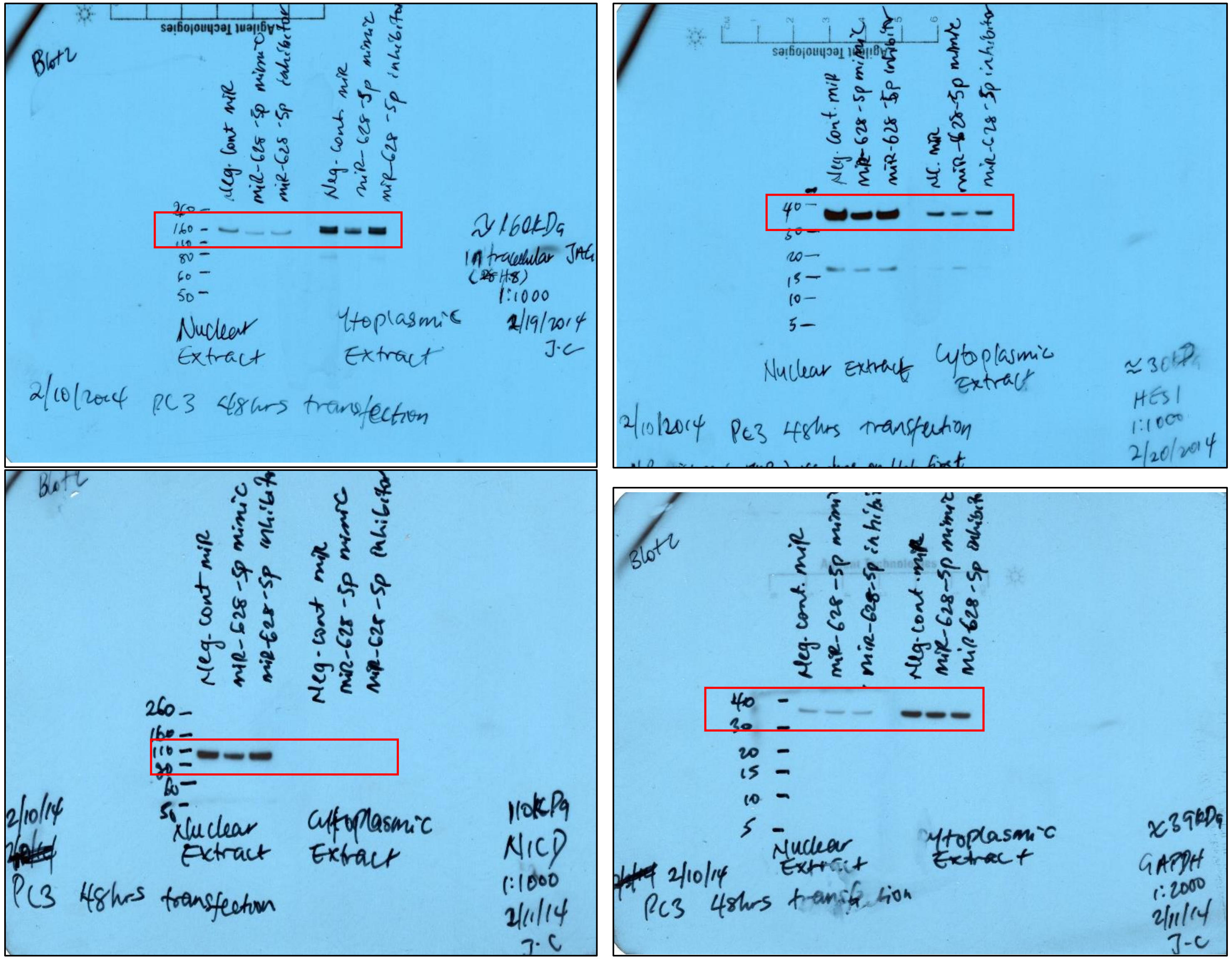

Full length images of blots for Fig. 5F in main paper. Red rectangles indicate the regions used in the figures. PVDF membranes were used for probing with the respective primary antibody. Film was utilized to develop membranes.

Figure 5

G

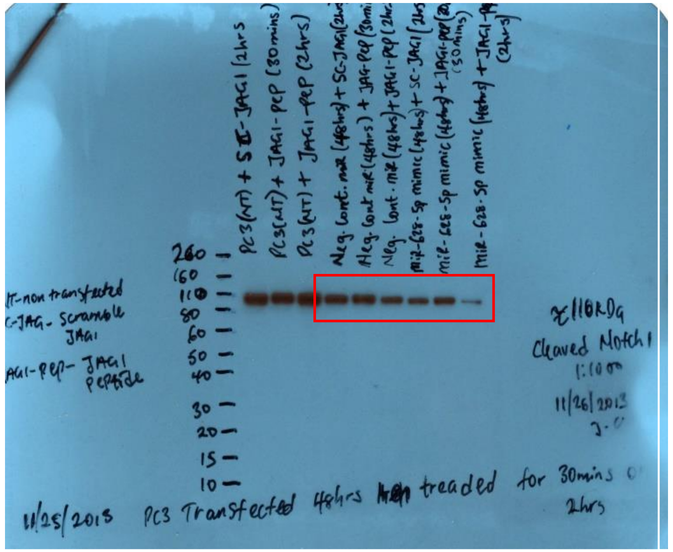

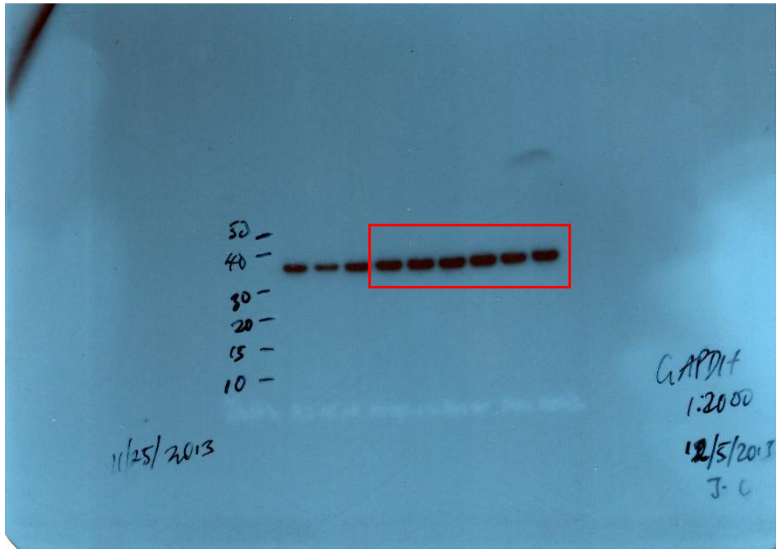

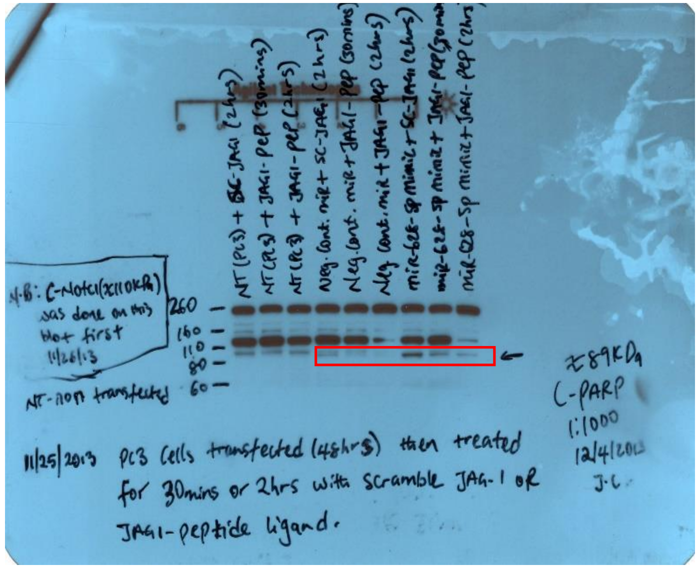

Full length images of blots for Fig. 5G in main paper. Red rectangles indicate the regions used in the figures. PVDF membranes were used for probing with the respective primary antibody. Film was utilized to develop membranes.

Figure 6

E

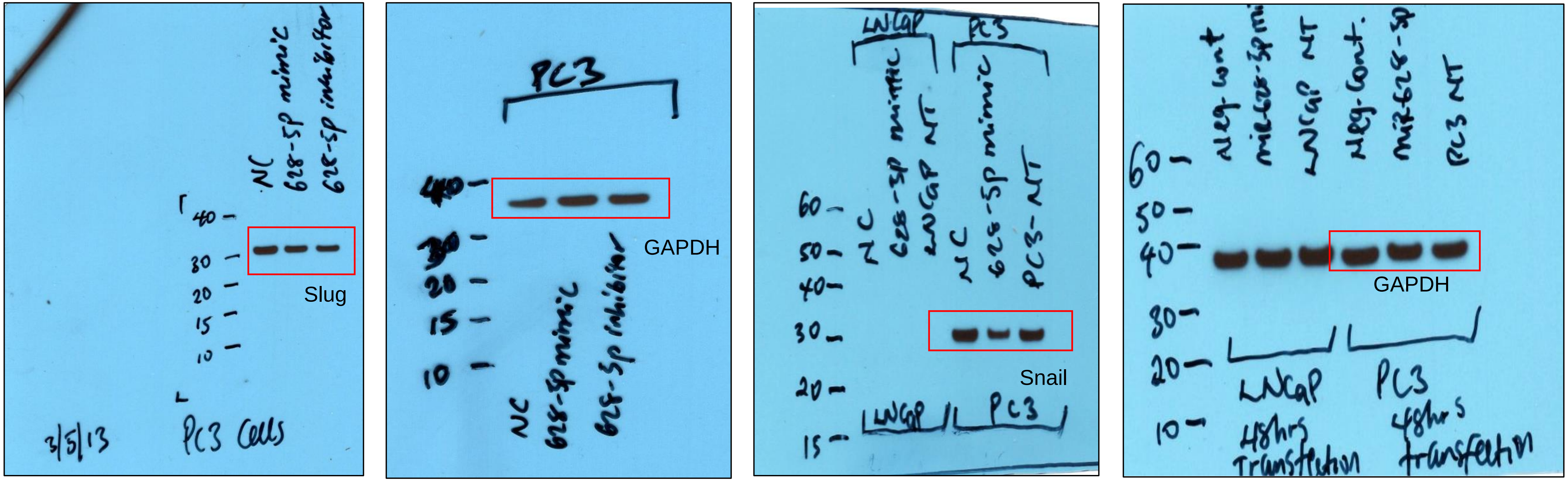

Full length images of blots for Fig. 6E in main paper. Red rectangles indicate the regions used in the figures. PVDF membranes were used for probing with the respective primary antibody. Film was utilized to develop membranes.

Figure 6

F

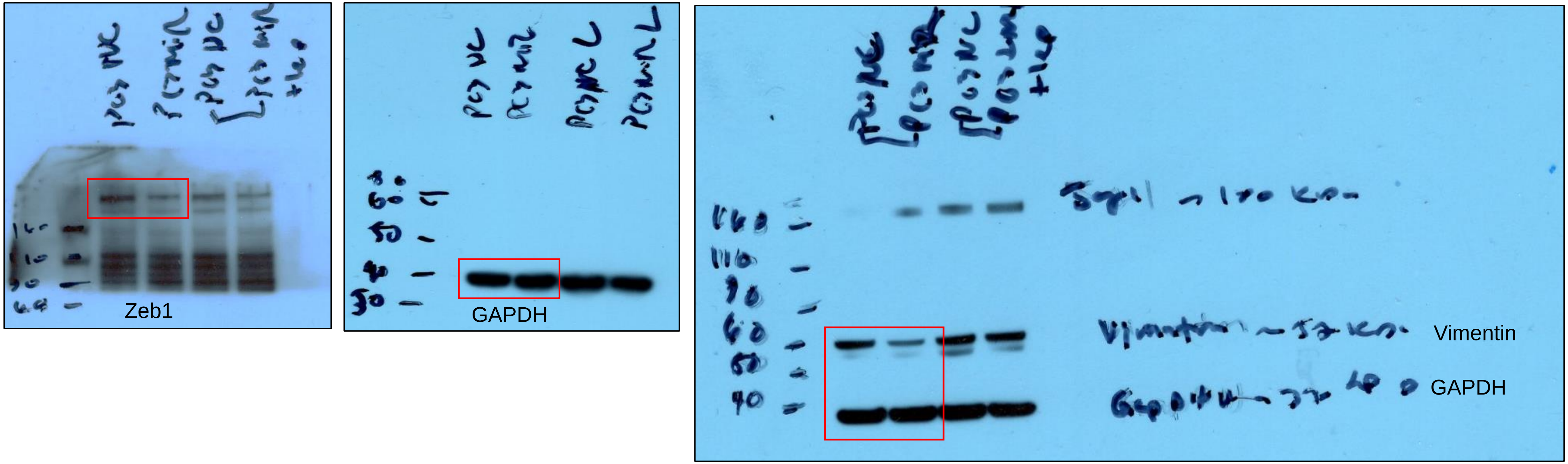

Full length images of blots for Fig. 6F in main paper. Red rectangles indicate the regions used in the figures. PVDF membranes were used for probing with the respective primary antibody. Film was utilized to develop membranes.

Figure 6

H- left and right

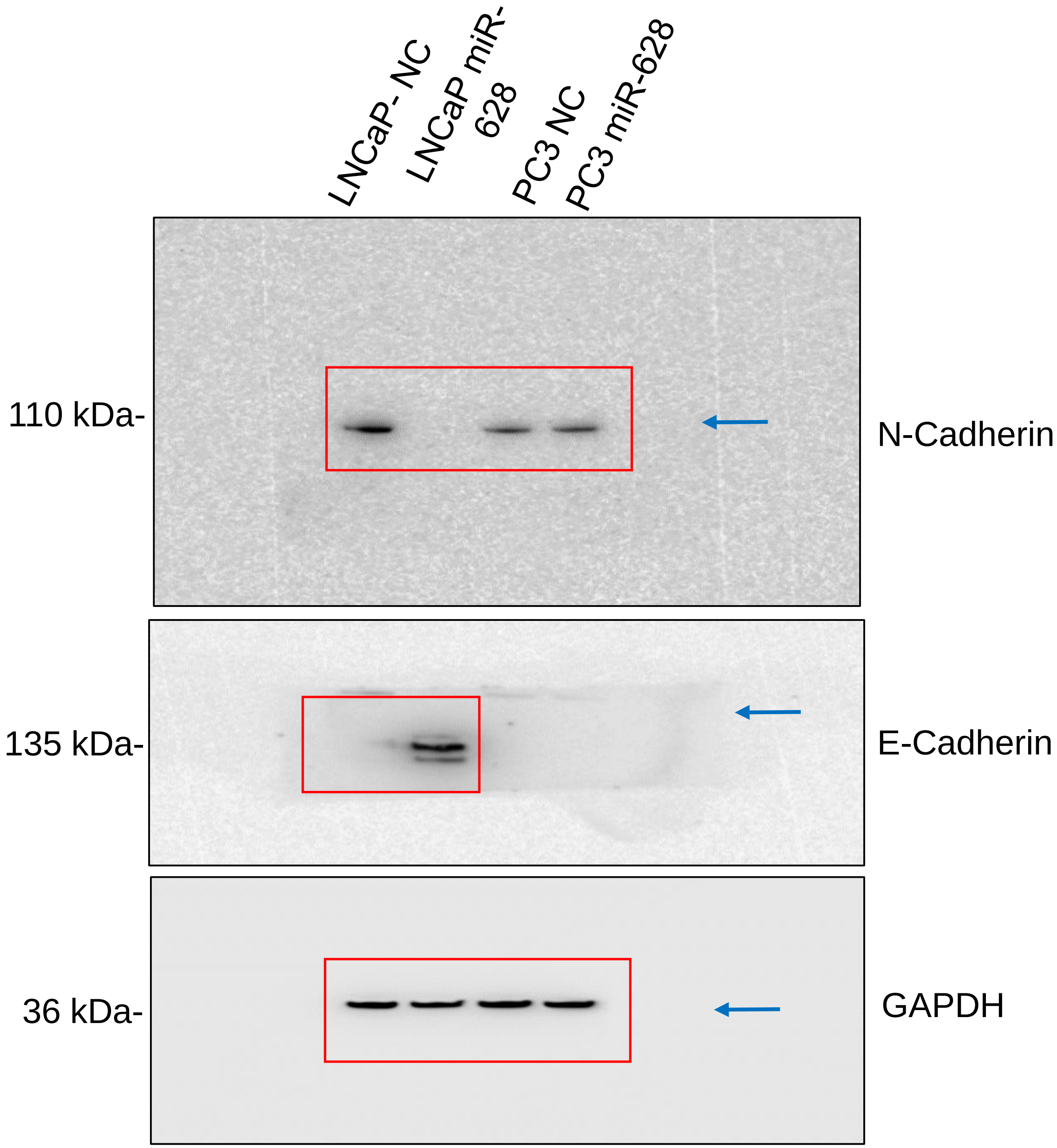

** membrane was cut previous to probing using molecular weight marker as reference. The Azure c500 was used to scan membranes.

Full length images of blots for Fig. 6H in main paper. Red rectangles indicate the regions used in the figures. PVDF membranes were cut where indicated using molecular weight marker as reference, and used for probing with the respective primary antibody. The Azure c500 was used to scan membranes.

Figure 6

H -right

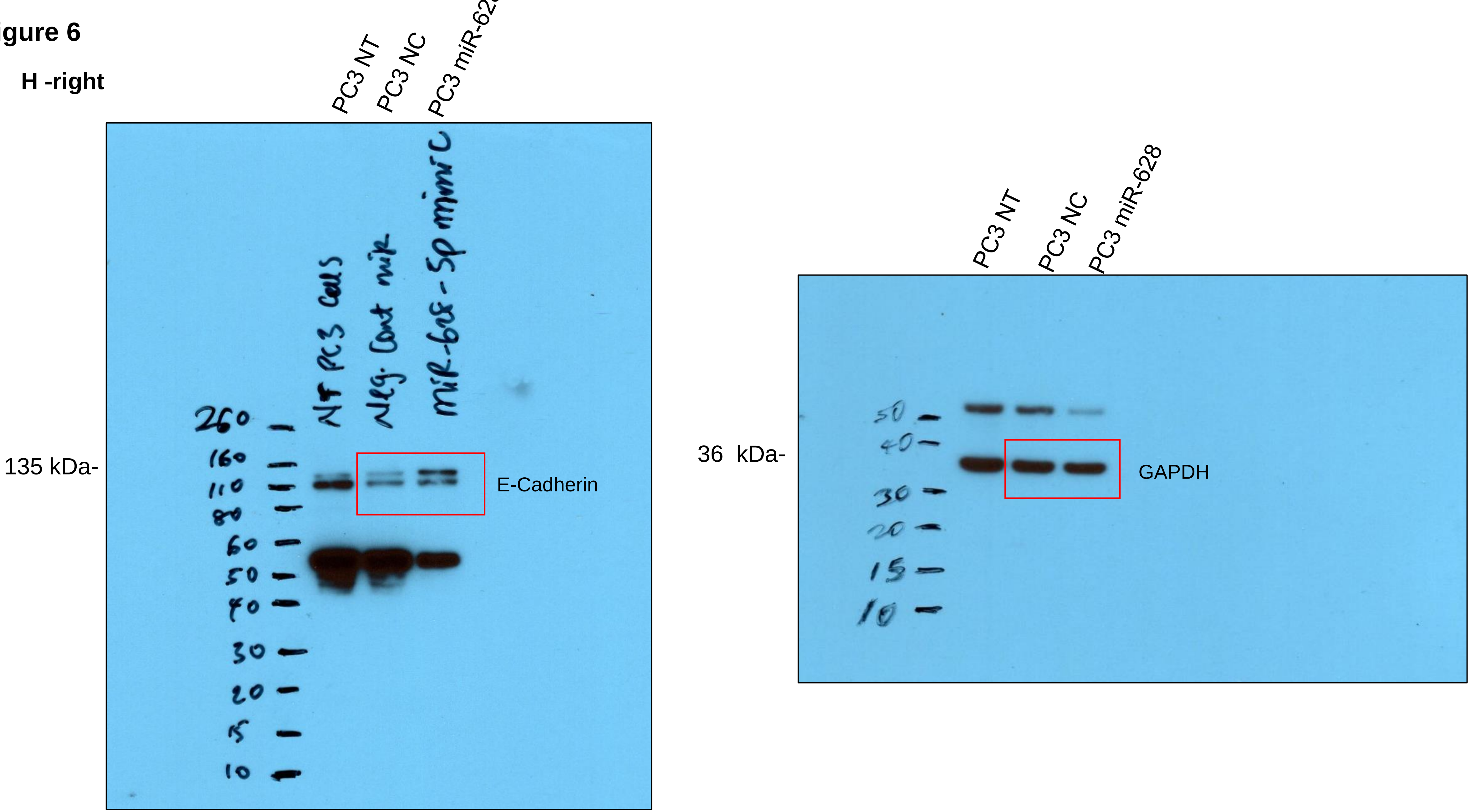

Full length images of blots for Fig. 6H in main paper. Red rectangles indicate the regions used in the figures. PVDF membranes were used for probing with the respective primary antibody. Film was utilized to develop membranes.

Figure 7

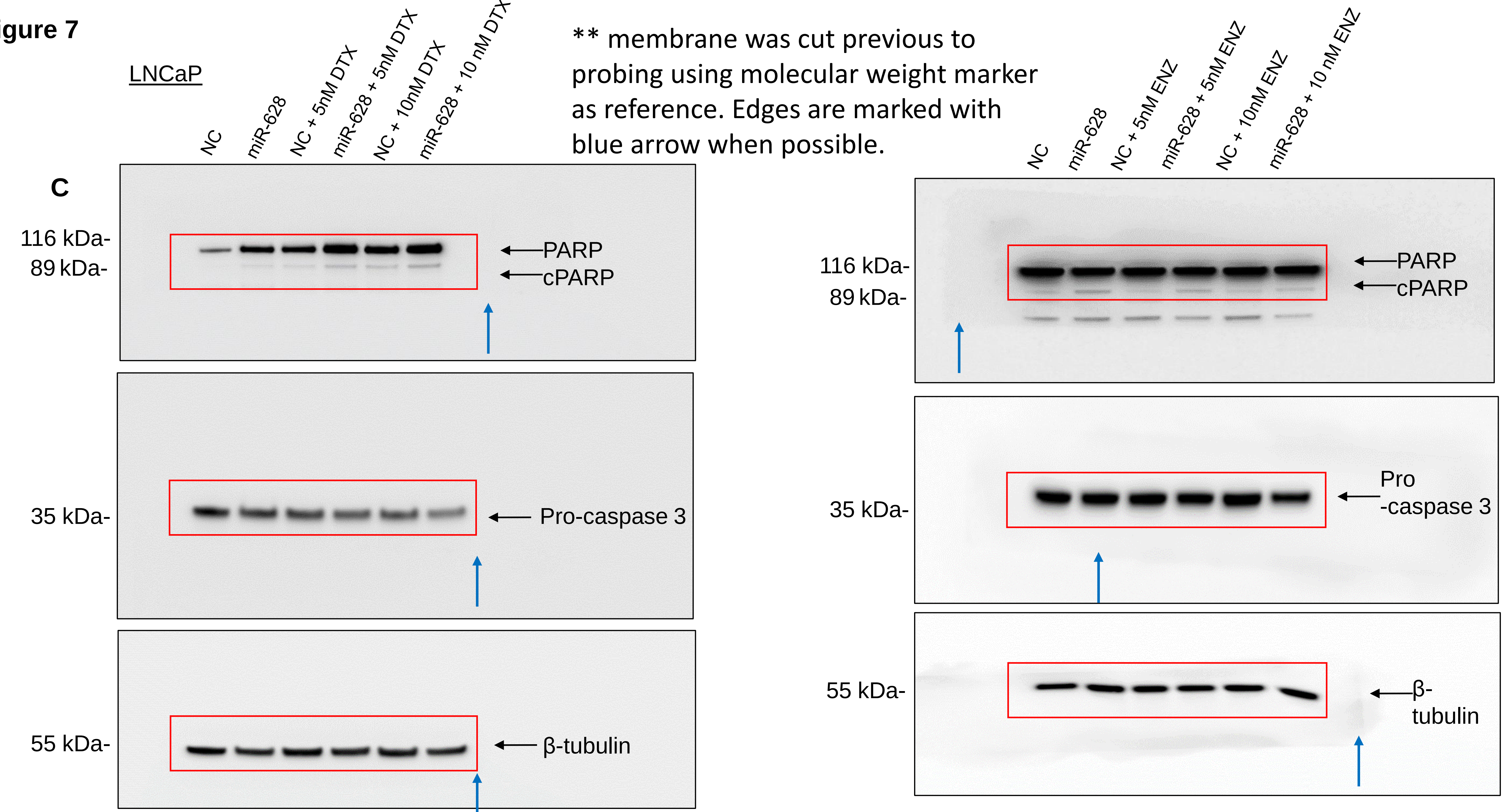

Full length images of blots for Fig. 7C in main paper. Red rectangles indicate the regions used in the figures. PVDF membranes were used for probing with the respective primary antibody. The Azure c500 was used to scan membranes.

Figure 3S

** membrane was cut previous to probing using molecular weight marker as reference. Edges are marked with blue arrow when possible.

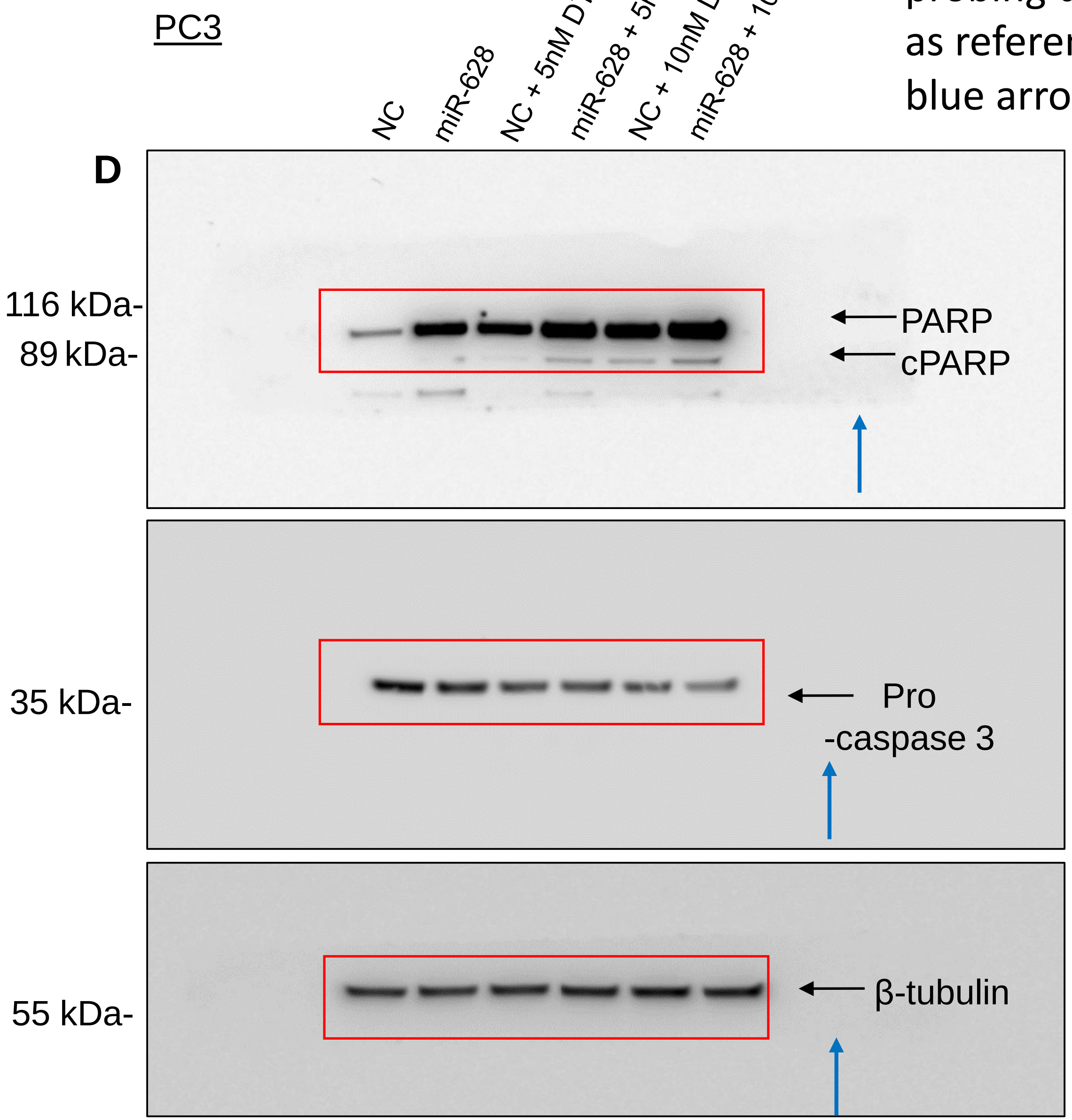

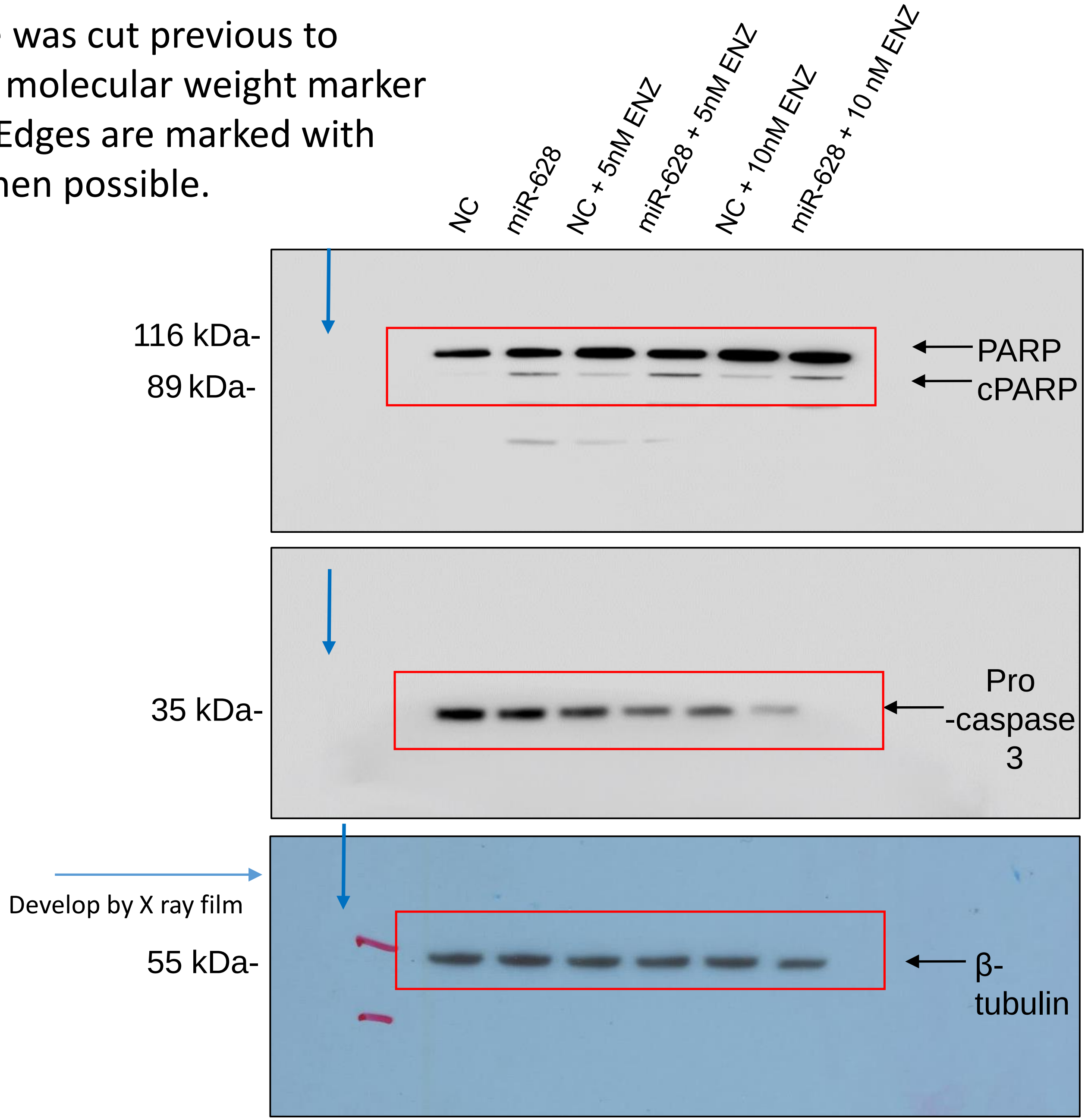

Full length images of blots for Fig. 7D in main paper. Red rectangles indicate the regions used in the figures. PVDF membranes were used for probing with the respective primary antibody. Film and the Azure c500 was used to develop membranes.
