# Supplementary material for: Leptin modulated microRNA-628-5p targets Jagged-1 and inhibits prostate cancer hallmarks
Source: Sci Rep. 2022 Jun 16;12:10073. doi: 10.1038/s41598-022-13279-x (PMC9203512; doi:10.1038/s41598-022-13279-x)
Supplement: Supplementary file 5 — Supplementary Information 5. [file 41598_2022_13279_MOESM5_ESM.docx]

**Supplementary figure legends**

***Figure 1S. MiR-628 is downregulated in PCa patient’s clinical samples compared to normal controls.*** miR-628 expression was assessed utilizing the TCGA-PRAD, normal (N=52, blue) vs. tumor tissues (N=494, orange) (*p<0.02).

***Figure 2S. JAG1 is overexpressed in PCa patient samples compared to a normal matched control.*** JAG1 expression was assessed utilizing the GSE41969 dataset and analyzed using the GEO2R web tool in matched benign (light purple circles, n=163) and PCa samples (dark purple squares, n=639) obtained from AA and CA men.

***Figure 3S. Overexpression of miR-628 and treatment of Enzalutamide or DTX decreases cell proliferation and induces apoptosis in PC3 cells.* (A)** PC3 cells transfected with miR-628 mimic or NC were treated with enzalutamide (ENZ) (5µM, or 10µM) or docetaxel (DTX) (5nM or 10nM). Cell viability was measured after 24h and 48h after transfection using MTT assay (*p<0.05, **p<0.01, ***p<0.001). **(B)** The effect of miR-628 and co-treatment of ENZ or DTX on PCa cell apoptosis was assessed through cleaved-PARP and pro-Caspase-3 expression using western blotting as described in the materials and methods section. Membranes were cut before hybridization using the molecular weight marker as a reference and imaged using an Azure imaging system. All western blot full images are provided in supplementary information files.

***Supplementary Table 1: Forward and reverse primers sequences used in the current study for gene expression.***
