# Supplementary material for: Leptin modulated microRNA-628-5p targets Jagged-1 and inhibits prostate cancer hallmarks
Source: Sci Rep. 2022 Jun 16;12:10073. doi: 10.1038/s41598-022-13279-x (PMC9203512; doi:10.1038/s41598-022-13279-x)
Supplement: Supplementary file 6 — Supplementary Information 6. [file 41598_2022_13279_MOESM6_ESM.docx]

**Leptin modulated microRNA-628-5p targets Jagged-1 and inhibits prostate cancer hallmarks**

**Leslimar Rios-Colon^1,2*^, Juliet Chijioke^1*^, Suryakant Niture^1^, Zainab Afzal^1^, Qi Qi^1^, Anvesha Srivastava^1^, Malathi Ramalinga^1^, Habib Kedir^1^, Patrice Cagle^1^, Elena Arthur^1^, Mitu Sharma^2^, John Moore^1^, Gagan Deep^2,3,4^ Simeng Suy^5^, Sean P Collins^5^, and Deepak Kumar^1**^**

**Supplementary Table 1: Forward and reverse primers sequences used in RT/qPCR assay.**

| **Gene** | **Forward and reverse sequences** |
| --- | --- |
| GAPDH | Forward 5’- CCACCCAGAAGACTGTGGAT – 3’  Reverse 5’-GTTGAAGTCAGAGGAGACCACC – 3’ |
| JAG1 | Forward 5’-tgggactgggacaccgatac – 3’  Reverse 5’- agtggcgctgtagtagttctc – 3’ |
| ZEB1 | Forward 5’-TTACACCTTTGCATACAGAACCC – 3’  Reverse 5’- TTTACGATTACACCCAGACTGC – 3’ |
| E-CADHERIN | Forward 5’- CGGAGAAGAGGACCAGGACT– 3’  Reverse 5’- GGTCAGTATCAGCCGCTTTC – 3’ |
| N-CADHERIN | Forward 5’- CGAATGGATGAAAGACCCATC C – 3’  Reverse 5’- GGAGCCACTGCCTTCATAGTCAA – 3’ |
| SNAIL | Forward 5’- aggttggagcggtcagc– 3’  Reverse 5’- ccttctctaggccctggct – 3’ |
| SLUG | Forward 5’ TTCGGACCCACACATTGCCT– 3’  Reverse 5’- GCAGTGAGGGCAAGAAAAAG – 3’ |
